# Commercial determinants of health—a scoping review of research ‘made in Germany’

**DOI:** 10.1093/eurpub/ckag030

**Published:** 2026-03-17

**Authors:** Kerstin Sell, Stefanie Nigg, Anna Leibinger, Stephan Voss, Carmen Klinger, Eva Rehfuess

**Affiliations:** Institute for Medical Information Processing, Biometry and Epidemiology, Chair of Public Health and Health Services Research, Faculty of Medicine, LMU Munich, Munich, Germany; Pettenkofer School of Public Health, Munich, Germany; Institute for Medical Information Processing, Biometry and Epidemiology, Chair of Public Health and Health Services Research, Faculty of Medicine, LMU Munich, Munich, Germany; Pettenkofer School of Public Health, Munich, Germany; Institute for Medical Information Processing, Biometry and Epidemiology, Chair of Public Health and Health Services Research, Faculty of Medicine, LMU Munich, Munich, Germany; Pettenkofer School of Public Health, Munich, Germany; Institute for Medical Information Processing, Biometry and Epidemiology, Chair of Public Health and Health Services Research, Faculty of Medicine, LMU Munich, Munich, Germany; Pettenkofer School of Public Health, Munich, Germany; Institute for Medical Information Processing, Biometry and Epidemiology, Chair of Public Health and Health Services Research, Faculty of Medicine, LMU Munich, Munich, Germany; Pettenkofer School of Public Health, Munich, Germany; Institute for Medical Information Processing, Biometry and Epidemiology, Chair of Public Health and Health Services Research, Faculty of Medicine, LMU Munich, Munich, Germany; Pettenkofer School of Public Health, Munich, Germany

## Abstract

Commercial products such as tobacco, alcohol, ultra-processed food and fossil fuels drive the global burden of non-communicable diseases (NCDs) and the escalating climate crisis. The concept ‘commercial determinants of health’ (CDOH) offers a framework for understanding the ways in which commercial actors, processes, and products influence health. With most CDOH research originating from Anglo-Saxon countries, we sought to map Germany’s CDOH research landscape and related scientific discourse. We conducted a scoping review according to a pre-registered protocol. Records were identified through systematic searches in Medline, Embase, Web of Science, and Google Scholar, last updated 6 December 2024, and by searching seminal CDOH literature. We included peer-reviewed articles (co-)authored by researchers affiliated with German institutions, which examined the public health effects of corporate sector practices; results were presented in an evidence map. We included 136 articles, comprising 64 original research articles (47.1%), 36 overview type articles (26.5%), and 17 opinion pieces (12.5%). Fifteen mentioned the ‘commercial determinants of health’ (11.0%). Research activities focused on the tobacco, alcohol, food, and pharmaceutical industries; articles were primarily concerned with political, scientific, marketing, and reputational management practices. A supplementary social network analysis showed fragmented authorship networks. CDOH are key upstream determinants to consider in the prevention of NCDs. Germany faces a substantial and growing burden of disease from NCDs but the country’s research on the CDOH is limited. We suggest that researchers embrace the scholarship on CDOH, and that practitioners harness relevant insights in addressing the commercially driven NCD burden.

## Introduction

Research on the commercial determinants of health (CDOH) has been fundamental in exposing the drivers of the substantial global burden of disease from non-communicable diseases (NCDs), and unmasking the strategies behind concerted efforts of corporate actors to oppose health-enhancing regulation and intervention.

CDOH have been defined as the ‘systems, practices, and pathways through which commercial actors drive health and equity’ [1]. In principle, this neutral definition accounts for the positive contribution of commercial actors and products on health, but its authors describe a system which is inadequately regulated and thus currently skewed towards a health-harming ‘pathological’ state [1]. The concept CDOH was only coined in 2012/2013 [2–4], popularized in 2016 [5] and 2022/2023 [6,7], and is now increasingly used; including in the World Health Organization’s (WHO) work in the area of NCDs [8,9]. CDOH research broadly encompasses three areas: products (‘unhealthy commodities’) or industries, corporate sector practices, and global drivers of ill health [10].

Just four **commercial products** constitute the main risk factors for NCD-related deaths: alcohol, tobacco, ultra-processed food and beverages, and fossil fuels. Along with occupational practices, these risk factors are responsible for an estimated 2.7 million deaths annually in the European Region, translating into 24.5% of all deaths [9]. The CDOH are also widely recognized as the driving force of the escalating planetary health and climate crises [11,12]. Fossil fuels have enabled some populations to lead ‘once unimaginable ways of living’ but continue to accelerate global warming and the break-down of eco-systems [12] with their associated impacts on human health, well-being, and equity. Further **industries** considered in CDOH research include gambling [13], firearms [14], automotive [15], news media [16], healthcare [17], finance [18], and pharmaceutical industries [19]. Multi- or transnational corporations are often–but not always–responsible for these products and activities [20].

**Corporate sector practices** describe how commercial entities affect health, i.e. through political, scientific, marketing, financial, labour and employment, supply chain, production and waste, and reputational management practices [1]. These are utilized across various health-harming industries [21–27]. Notably, abundant research on corporate political practices partly predates the popularization of CDOH as a concept [2,28–33]. Examples include lobbying, bribery, litigation [1]; revolving doors, campaign and party donations, direct participation in government committees, influence on international trade regulations, and tied aid [30,33]. Public–private partnerships can constitute a corporate political practice, giving corporations access to public resources and regulatory influence [34,35]. The disproportionate policy-making influence of corporations has been described as a ‘corruption of democracy, not an element of participatory democracy’ [33].

**‘Global drivers’** of the CDOH are linked to neoliberalism as the political-economic philosophy dominating in the Western world and resulting in global economic integration, trade and investment liberalization, de-regulation, decreasing state power, and the rise of monopolistic transnational corporations [1,36]. This consolidation of corporate power is evidenced by the large number of transnational corporations among top economies [18,37]. Recent work has highlighted the harmful impact of financialization on public health, which is driven by the increasing role of financial actors and institutions in economies [18], including in healthcare services [38].

CDOH research has thus gained substantial traction and research agendas for the field have been formulated [19,39,40,41] (further references in Supplementary Material 1). CDOH provides a concept, a set of methods [42], and a label which facilitates identification of relevant research and which is crucial for building a multidisciplinary scientific community [5]. In a heterogeneous field, we argue that this label strengthens a joint identity, synergies, and visibility. With its fundamental insights into the upstream drivers of ill health, CDOH research is a promising area of scientific enquiry to support the Sustainable Development Goals and public and planetary health efforts.

To date, much of the research on CDOH, particularly conceptual work, originates from the United Kingdom, the United States, and Australia [1,7,11,24,43]. However, it is as relevant in Germany to integrate a CDOH perspective in research on NCDs: In comparison with other countries with equal or lower health expenditure, Germany’s life expectancy has been deemed ‘disappointing’ [44] – which appears to be caused by glaring deficits in the prevention of NCDs and efforts to tackle their underlying risk factors [44,45]. Compared to other European countries, Germany ranks very low in the regulation of tobacco, alcohol, and unhealthy foods and beverages [46]. This is likely linked to the influence of powerful industry lobbies and limited lobbying regulation in the country [47]. Public health institutions in Germany rarely refer to such structural drivers of health problems, and health-related research in the country is dominated by biomedical rather than population-level research. The country’s federalist system further hinders country-wide health regulation [45]. Given Germany’s strong economic and political role in Europe and beyond, we identify an urgent need to obtain a better overview of CDOH research conducted in Germany.

### Objectives

We aimed to systematically identify and map CDOH research and the related scientific discourse authored or co-authored by researchers based in Germany. We sought to identify research fields, networks, and CDOH focus areas in public health and other disciplines in the country.

## Methods

### Research approach and concept

We conducted a scoping review as outlined in our protocol (https://osf.io/6pxzt) [48]. We followed the reporting standards for scoping reviews, PRISMA-ScR [49].

Gilmore and colleagues’ (2023) CDOH framework illustrates the multi-level commercial influences on population health and describes how corporate sector practices influence political, economic and social systems [1,50]. The authors describe seven corporate sector practices (see above), which we drew on to operationalize our research question and define eligibility criteria.

### Data sources, search strategies

Our review consisted of two strands [48]. In strand 1, we conducted forward citation searching of seminal literature, identified in a recently published CDOH textbook [7]. This textbook contained 1,289 non-unique references, and we included 134 references with over 50 citations (as per Scopus metrics [51]) as seminal, alongside articles in the Lancet Commission series on CDOH [1,20,11], relevant publications by seminal authors, and results from a targeted Google Scholar search. We drew on the TRACiS statement recommendations for citation searching [52], conducted forward citation searching in Scopus [51], and filtered results by ‘Germany’ on 04/08/2024. In strand 2, we searched Medline and EMBASE (via OVID), Web of Science, and Google Scholar on 29/07/2024. Our search strategy was reviewed by an information specialist and consisted of two blocks: (i) German affiliation; and (ii) commercial or corporate determinants or practices.

We conducted a search update on 04/12/2024 to include articles from a special issue on the commercial determinants in *Health Promotion International* and to consider additionally relevant search terms identified through our review; these were added to the original searches (all searches available in Supplementary Material 2).

### Eligibility

We included articles

- with at least one author affiliated with an institution in Germany;
- which examine corporate sector practices and their effects on the political, economic, and social systems affecting health from a critical stance, or explicitly mention CDOH;
- are peer-reviewed; and
- are written in English or German.

We did not include any industry-sponsored research, nor research exclusively authored by industry-affiliated individuals. Details are available in our methodological appendix (Supplementary Material 3).

### Data selection and extraction

Records identified in the two search strands were de-duplicated in Zotero [53] and titles and abstracts subsequently screened for eligibility in Rayyan [54]. An initial subset of 320 articles (12%) were screened in duplicate to calibrate screening. We conducted full text screening individually after screening 38 full texts in duplicate (6%). Unclear cases were discussed between the two first authors until consensus was reached.

After trialling the Microsoft Excel-based extraction sheet, data extraction was undertaken by one researcher with thorough review by a second researcher. Beyond basic article information, we extracted information on publication type, scientific discipline, geographic focus, industry actor(s), corporate sector practice(s), funding, and conflict of interest (COI) information. We also performed a simple keyword search in the full text of all included studies to identify if the term ‘CDOH’ was mentioned.

For data extraction of corporate sector practices, funding, and COI, we used abridged methods of qualitative content analysis, informed by Schreier [55]. To this end, we drew on examples of corporate sector practices [1] to inform our basic coding frame. We applied existing categories deductively and developed labels for sub-categories inductively (Supplementary Material 3).

### Data charting and analysis

We developed an evidence map [56] and cross-tabulated (i) corporate sector practices (i.e. political, scientific, marketing, financial, labour and employment, supply chain, production and waste, and reputational management practices) and (ii) industries (i.e. tobacco, alcohol, food, fossil fuel, pharmaceutical, healthcare, health technology, gambling, chemical) examined in the included articles.

Additionally, we undertook a social network analysis (SNA) to supplement the scoping review, as recommended by Cowhitt [57]. We cleaned bibliometric data from included articles and imported them into VOSviewer [58], using the software’s function to illustrate co-authorship networks. We considered all authors for whom a minimum of two articles were included in our review, employed full counting, and included all clusters and authors (Supplementary Material 3). Identified clusters of authors were subsequently inspected visually, and underlying publications reviewed to determine the countries of the authors’ affiliations and to identify focus areas of SNA clusters.

### Ethics

The ethics commission of the LMU Munich’s Faculty of Medicine attested that this work did not require ethics review (24-0652-KB).

## Results

**Figure 1.**
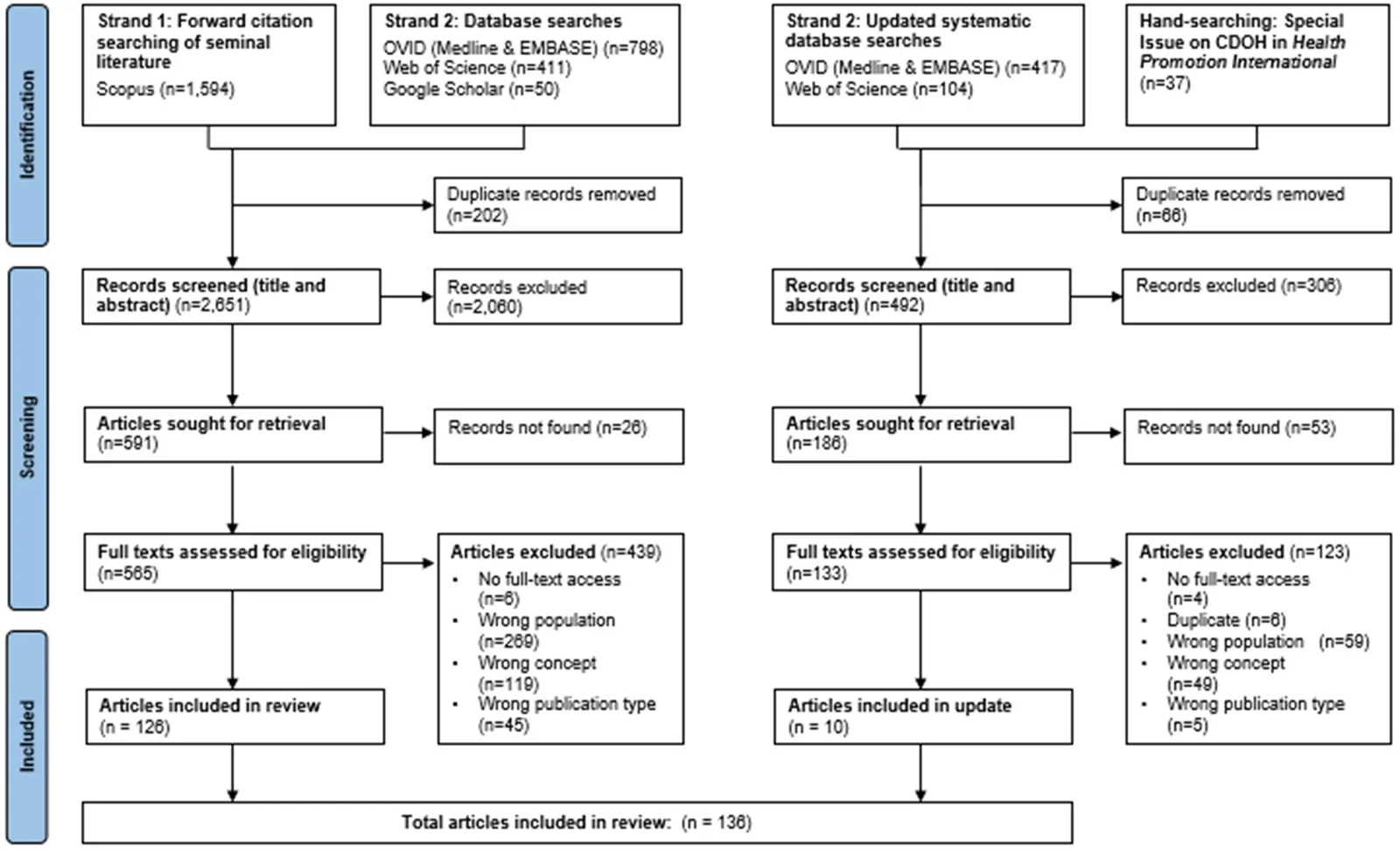
Flowchart including search update.

Database searches and forward citation searches returned 2,853 records. After de-duplication, we screened 2,651 records (including seminal literature) and included 591 records for full-text screening. We eventually included 126 records. In our search update, we screened 492 records and included 10 additional articles, hence including a total of 136 records (Fig. 1/ Supplementary Material 4).

### Characteristics of included studies

The majority of included articles were published from 2020 onwards (*n* = 87, 64%). We categorized the discipline of 91 records as public health (66.9%), 30 as healthcare (22.1%), and 15 as ‘other’ (e.g. ethics, political science) (11.0%). Public health nutrition was the most common sub-discipline (*n* = 10, 7.4%).

Around half of the included records were original research, comprising primary research (*n* = 52, 38.2%) and systematic reviews (*n* = 12, 8.8%). Just over half of all articles (58.1%) had a specific geographic focus, primarily on Germany (*n* = 44, 32.4%), Europe (*n* = 8, 5.9%), and the United States (*n* = 4, 2.9%) (Table 1/ Supplementary Material 4). The remainder (41.9%) had no specific geographic focus.

**Table 1.** Characteristics of included articles

Category	Sub-category	*n* (%)
Publication year	Before 2010	11 (8.1)
	2010 - 2019	38 (27.9)
	2020 - 2024	87 (64.0)
Scientific discipline	Public health	91 (66.9)
	Healthcare	30 (22.1)
	Other	15 (11.0)
Type of article	Original research, qualitative	22 (16.2)
	Original research, mixed methods	16 (11.8)
	Original research, quantitative	14 (10.3)
	Original research, systematic review	12 (8.8)
	Overview article^a^	36 (26.5)
	Opinion piece	17 (12.5)
	Theoretical work	6 (4.4)
	Other	13 (9.6)
Geographic focus	No specific country focus	57 (41.9)
	Germany	44 (32.4)
	Europe (EU/WHO region/continent)	8 (5.9)
	United States	4 (2.9)
	Low- and middle-income countries	4 (2.9)
	Other	19 (14.0)
Industry/sector/product^b^	Food^c^	50 (36.8)
	Pharmaceutical	43 (31.6)
	Tobacco	30 (22.1)
	Alcohol	23 (16.9)
	Healthcare	17 (12.5)
	Health technology	10 (7.4)
	Gambling	7 (5.1)
	Chemical	6 (4.4)
	Other^d^	23 (16.9)
Corporate sector practice^e^	Marketing	75 (55.1)
	Scientific practices	63 (46.3)
	Corporate political activity	42 (30.9)
	Reputational management	39 (28.7)
	Financial practices	25 (18.4)
	Supply chain, production and waste	17 (12.5)
	Labour and employment	7 (5.1)
	Other^f^	19 (13.9)
CDOH mentioned?	Yes	17 (12.5)
	No	119 (87.5)
Funding source stated?	Yes	84 (61.8)
	No	52 (38.2)
Funder categories^g^	No specific funding	22 (26.2)
	Public funding	43 (51.2)
	Charity	9 (10.7)
	Other	2 (2.4)
COI stated?	Yes	111 (81.6)
	No	25 (18.4)
COI categories^h^	None	72 (64.3)
	Financial	25 (22.5)
	Non-financial	20 (17.9)

a Often unsystematic literature reviews or introduction to the topic without a methods section.

b Sum of percentages exceeds 100% as most articles have examined multiple industries.

c Food industry including non-alcoholic beverage industry.

d ‘Other’ industries included the fossil fuel (*n* = 4), asbestos (*n* = 4), mining (*n* = 3), finance (*n* = 3), IT/technology (*n* = 3), social media (*n* = 2), and car/transportation industries (*n* = 2).

e Sum of percentages exceeds 100% as most articles have examined multiple corporate sector practices.

f Other corporate sector practices included health-harming data use practices, e.g. by sharing individually-identifying data with advertisers [22,139–141] or legal practices, primarily litigation [98,142,143].

g Percentages calculated based on the 84 articles which provided a funding source.

h Percentages calculated based on the 111 articles which provided a COI statement. Sum of percentages exceeds 100% as some articles stated financial and non-financial COIs.

### Industries

Among the 136 articles authored or co-authored by individuals at German institutions, the **food (and non-alcoholic beverages) industry** was most frequently examined (*n* = 50, 36.8%) (Table 1), in particular, the products sugar/sugar-sweetened beverages (*n* = 22, 16.2%) and baby food/commercial milk formula (*n* = 10, 7.4%). Examples include analyses of the food retailer Lidl’s self-regulation pledge [59] and of marketing claims on infant feeding bottles [60]. The **pharmaceutical industry** was the second most frequently examined industry (*n* = 43, 31.6%). Examples comprise a review about the ‘Sisi-Syndrome’, a non-existent form of depression fabricated to expand the market for antidepressants [61], and a qualitative analysis of orphan drugs product placement in the television series ‘House MD’ [62]. The **tobacco industry** was the third most frequently examined industry (*n* = 30, 22.1%). Examples include research examining and refuting the tobacco industry’s claims about tobacco smuggling [63] and an analysis of corporate political activity in tobacco control legislation in Ukraine [64]. The **alcohol industry** was examined in 23 articles (16.9%) and the healthcare and health technology industries in 17 (12.5%) and 10 (7.4%) articles, respectively. Very few articles examined **gambling** (*n* = 7) and **chemical** industries (*n* = 6). 39 articles (28.7%) assessed more than one industry.

### Corporate sector practices

**Marketing** and **scientific practices** were examined most commonly, in 75 (55.1%) and 63 (46.3%) articles, respectively (Table 1). For **marketing practices**, we mainly identified articles examining advertising strategies (*n* = 33, 24.3%), such as unhealthy foods targeted towards children [65]; and labelling practices (*n* = 12, 8.8%), such as ‘drink responsible’ messages. Examined **scientific practices** covered the examination of industry sponsorship or COIs on study outcomes (*n* = 29, 21.3%, *n* = 12, 8.8%, respectively), e.g. in surgical trials [66]; scientific misconduct (*n* = 24, 17.6%), e.g. alteration of data and omission of inconvenient research questions [67]. We furthermore categorized the disregard of evidence (*n* = 12, 8.8%) as a scientific practice to avoid governmental regulation, e.g. only recognizing evidence from randomized controlled trials as ‘valid’ [22].

For **political practices** (*n* = 42, 30.9%), we identified articles examining lobbying (*n* = 20, 14.7%), for example the sugar-sweetened beverage industry’s influence on governments through special interest groups [68], as well as industry self-regulation (*n* = 11, 8.1%), such as pharmaceutical industry voluntary disclosure of payments to physicians [69].

Under **reputational management practices** (*n* = 39, 28.7%), we identified articles examining collaboration with media institutions (PR-firms, newspapers, social media) [27,64,70] and ‘health-washing’, described as the ‘misuse of health to advance self-interest’ [71]. Under **financial practices** (*n* = 25, 18.2%), we included, for example, bribery of healthcare practitioners [69,72–83].

We considered ‘production’ as part of ‘**supply chain and waste management**’ corporate sector practices (*n* = 17, 12.5%) and identified articles assessing formulation [84,85], reformulation [86], aspects of the retail environment (‘obesogenic environment’) [87,88], and pollution [89–91].

Least discussed were **labour and employment practices** (*n* = 7, 5.1%), e.g. green tobacco sickness and child labour among tobacco workers and farmers in low- and middle-income countries [92].

Three articles did not describe any corporate sector practices [93–95] and 82 articles (60.3%) described more than one practice.

### CDOH label

We identified 15 articles explicitly referring to the **‘commercial determinants of health’** in the article’s full-text (11.0%) [5,23,60,71,84,90,93–101]. These were all published after 2016, when the concept was popularized [5]. The CDOH label was used in articles about tobacco, alcohol, and food industries but not in articles about the pharmaceutical and healthcare industries. Only two articles mentioning CDOH focused on Germany [60,97].

### Evidence map

**Figure 2.**
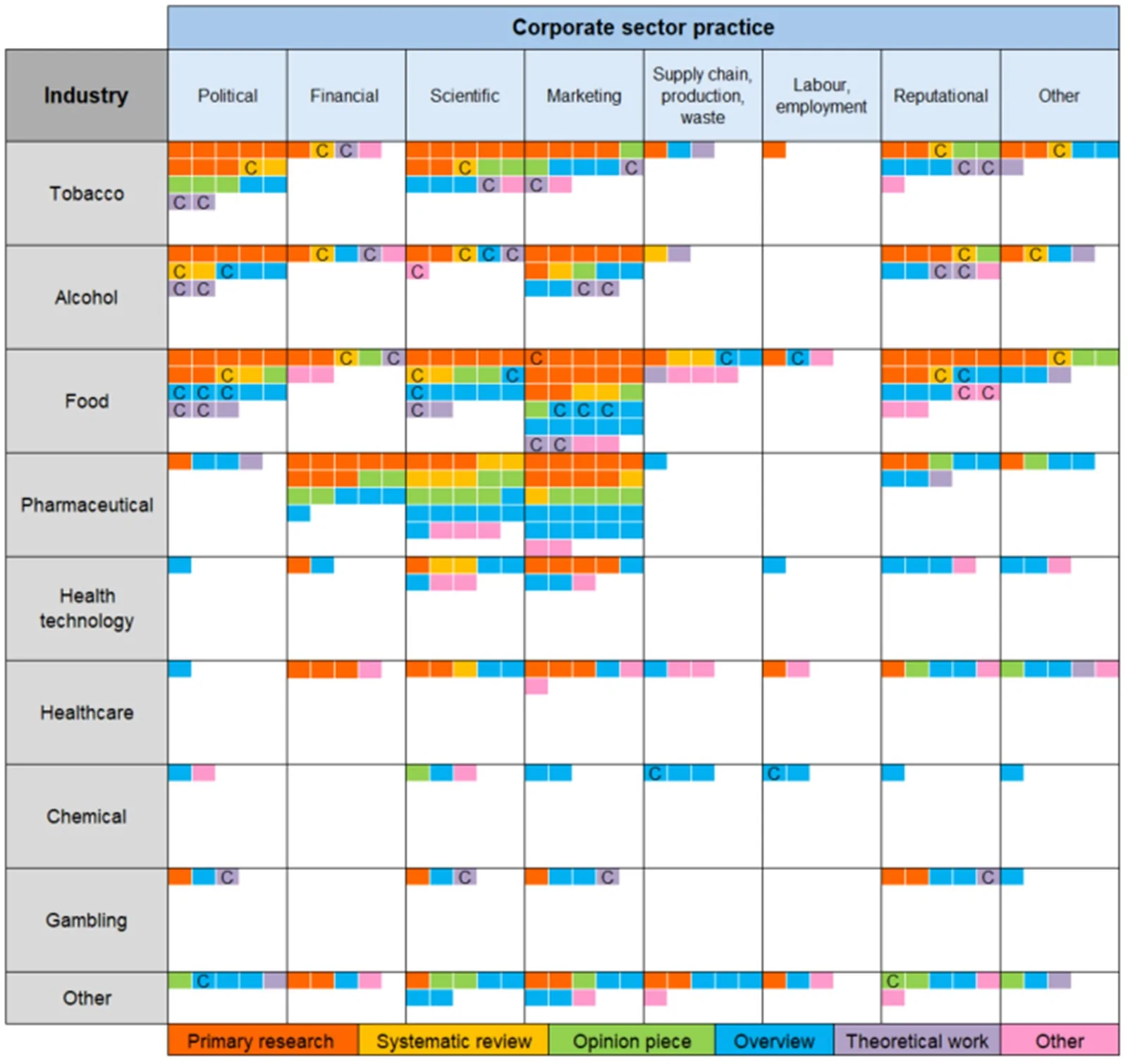
Evidence map of research on the commercial determinants of health originating from Germany or including co-authors based in Germany, showing corporate sector practices and industries. One small coloured cell represents one article mentioning the respective industry and corporate practice. One article may have resulted in multiple cells if it discussed multiple industries and/or corporate practices. Articles were omitted from the evidence map if they only used the CDOH label but did not describe any corporate sector practices [93–95]. Article type is indicated by colour (primary research—red, systematic review—orange, opinion piece—green, overview—blue, theoretical work–purple, other—pink), use of the term CDOH is indicated by the letter ‘C’.

The evidence map charts corporate sector strategies against key industries. It shows **three large clusters**: food industry marketing (*n* = 29), pharmaceutical industry marketing (*n* = 27), and pharmaceutical industry scientific practices (*n* = 24) (Fig. 2). Cluster 1 is mainly concerned with media advertisement for unhealthy or highly processed foods [5,23,26,65,68,88,97,101–105]. Cluster 2 primarily addresses the pharmaceutical industry’s approaches to convince medical doctors to prescribe their products [69,73–79,81–83,106–108]. Cluster 3 focuses on the influence of pharmaceutical industry funding on study results [61,66,80,83,109–119].

We further identified **six medium-sized clusters**: food industry corporate political practices (*n* = 18), alcohol industry political practices, tobacco industry political practices, food industry scientific practices, food industry reputational management (all *n* = 17), and pharmaceutical industry financial practices (*n* = 16).

### Social network analysis

**Figure 3.**
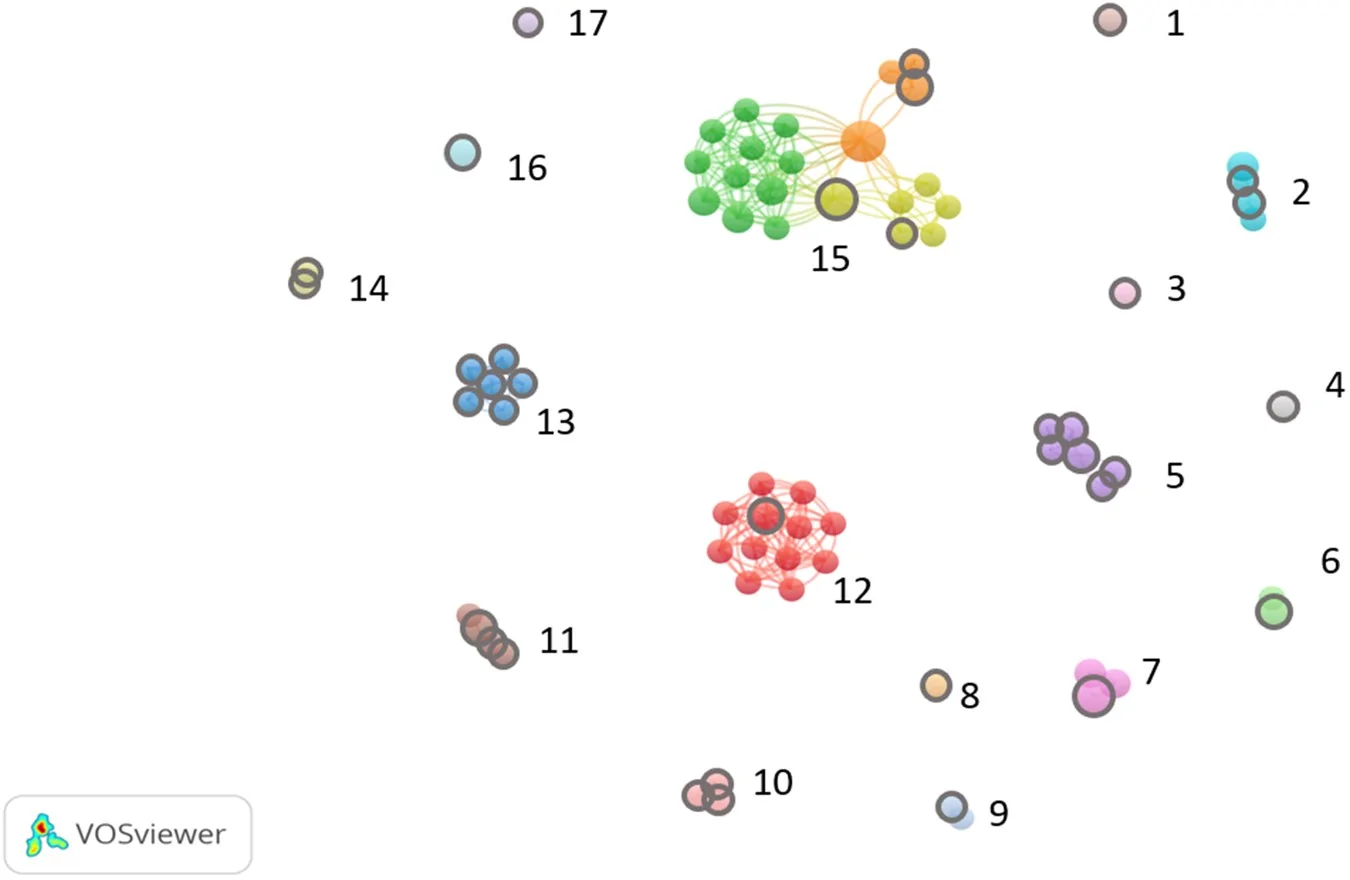
Social network analysis of research on the commercial determinants of health originating from Germany or including co-authors based in Germany, showing clustering of authors connected by co-authorship. Individuals who authored or co-authored two or more publications (*n* = 71) were included. Bubbles denote individual authors, links between bubbles denote co-authorship, grey circles denote authors based at German institutions (on at least one publication).

In the SNA, we identified 71 individuals who were authors on two or more publications. Their co-authorship networks formed seventeen isolated clusters of authors (Fig. 3). The number of authors in each cluster ranged from one (clusters 1, 3, 4, 8, 16 and 17) to twenty-one (cluster 15).

The two largest clusters were focused on CDOH, NCDs, and public health nutrition (cluster 15) and oral health (cluster 12). Only four authors in cluster 15 and only one author in cluster 12 were based at German institutions (Fig. 3). In the smaller clusters, the majority of authors were from German institutions.

In articles authored by cluster 2, 7, 12 and 15 the term CDOH was explicitly mentioned. These clusters tend to represent conceptual literature on CDOH, often examining multiple industries and corporate sector practices. The smaller clusters mostly focused on one product, industry, corporate sector practice or topic, e.g. asbestos (cluster 6), tobacco industry (cluster 11), pharmaceutical industry marketing (cluster 5), or COI in medical trials (cluster 13).

## Discussion

### Principal findings in context

We systematically mapped CDOH research originating from Germany or including co-authors based in Germany. Out of 136 included articles, only 15 used the label ‘CDOH’. We found that CDOH-related research from Germany is fragmented and mostly focused on four key industries and just four corporate sector strategies, i.e. marketing, scientific, political, and reputational management practices. Analysis of co-authorship networks showed two large clusters but otherwise largely disconnected groups of authors.

In a similar review, Burgess and colleagues (2024) studied the scope of CDOH research globally, identifying a substantial increase in CDOH literature in recent years, much of which consisted of conceptual literature and qualitative research [120], which matches our findings. Their review was, however, limited to literature using the term commercial (or corporate) determinants and did not focus on a single country.

### Industries covered

Our evidence map identified large bodies of CDOH research on the pharmaceutical as well as alcohol, tobacco, and ultra-processed food industries. The last three are commonly referred to as ‘unhealthy commodities’ industries, characterized by ‘high profit margins’ and easy accessibility for consumers [22]. Much CDOH research conducted internationally has focused on these industries as drivers of NCDs [20,120]. Other researchers have categorized gambling, breast milk substitutes, palm oil, fossil fuel, automobile, and mining industries as unhealthy commodities [1], all of which were examined in very few included articles.

Contrasting with the abovementioned review [120], we also found large clusters of research concerned with the pharmaceutical industry, disconnected from other clusters. This is indicative of a distinct research ‘world’ primarily concerned with COIs and not using the label ‘CDOH’.

Our SNA showed a disconnect between authors investigating distinct industries. This has been observed elsewhere in public health where ‘[t]obacco experts rarely speak to those in alcohol, nutrition, or sexual health, with no apparent recognition that, far from being unique and separate, the behaviours they all address comprise a typical Saturday night out for large sectors of the population [2].’ Such siloed work, including only few partners and disciplines, is common in some research fields, albeit mostly in the humanities [121], and tends to decrease as a research field matures [122]. The fragmented clusters identified in our analysis may thus reflect a young research field. It demonstrates that, at present, researchers do not examine cross-industry practices, thus missing out on systems-level and more structural insights. This is concerning, given that industry actors have been shown to cooperate across diverse industries [123].

### Corporate sector practices addressed

Articles examining **scientific practices** investigated the influence of industry sponsoring on research outcomes. A common venue of CDOH research, it has been suggested to expand this research by examining the influence of various industries on other structures of science, e.g. peer review, academic publishing, conferences, and guideline development [124].

**Political practices** included articles about lobbying, which is frequently examined in CDOH research on corporate political activity [32,33,125–129]. We also identified a number of articles examining industry self-regulation pledges, a strategy used by industry actors to prevent regulation [130]. We subsumed production-related practices including formulation under the **supply chain and waste practices** category, e.g. formulation of commercial baby foods with excessively high sugar content to influence sweet taste preference [131].

**Financial practices** were investigated in very few articles, financialization was only examined in one [132].

### CDOH label

The rare mentions of the ‘commercial determinants’ in included articles may partly be due to timing, with the term only becoming more widely used after 2016; diffusion of the concept may be slow. Partly, authors use similar terms, e.g. corporate or commercial influence [30]. These terms reflect an awareness of the relevance of structural commercial determinants–whilst authors may not be aware of the CDOH body of literature, which has been described as ‘confined to the health sciences’ [133].

Other authors may deliberately eschew referencing the CDOH literature or avoid the CDOH label for fear of their research being perceived as ‘ideological’, which may limit their credibility for providing policy advice [134]. Others may be deterred in light of the intimidation CDOH researchers experience [135,136]. These concerns should not be taken lightly. Whether the term CDOH is used or not, leveraging insights from this rich literature will be critical if we want to understand–and ultimately tackle–the structural determinants of NCDs.

### Strengths and limitations

Our review drew on a pre-registered protocol, from which we deviated minimally. In addition to CDOH *research*, we included opinion pieces as an important source of the related scientific discourse. Since we conducted our searches, a validated search filter for Germany has become available [137], which may work better than our non-validated filter. While we do not expect to have identified all CDOH research with co-authors from Germany we feel that our two-pronged search approach and the review update enabled us to find a large share of relevant research.

In undertaking this review, we had to define what research we considered to be ‘CDOH research’. We thus developed generic eligibility criteria that were applicable across the seven corporate sector practices and added more detailed criteria for specific practices. We found that occupational health and environmental research in principle constitute relevant CDOH fields. Given the size of these fields we decided to exclude them from this review, unless health effects linked to a specific industry were reported. Any research from these fields and using the CDOH label would, however, have been included.

While we only screened and extracted a subset of records in duplicate, we thoroughly calibrated our screening, and conducted multiple quality checks during data extraction.

Lastly, some of the included articles only had one co-author with a German affiliation, which means the included research is in fact only partially ‘made in Germany’.

## Conclusion

To our knowledge, this is the first attempt to map one country’s CDOH research landscape. We demonstrate that the internationally established research field ‘commercial determinants of health’ is less established in Germany. At the same time, there is great potential for building an impactful CDOH community in the country. We encourage researchers, research institutions, and funders to seize this opportunity, in order to strengthen NCD prevention and effectively tackle root causes.

Specifically, we recommend that **individual researchers** engage with the conceptual literature and the full spectrum of CDOH research, spanning multiple industries, corporate sector practices, and methodologies. Future work may address the identified evidence gaps in industries, notably gambling and car industries; and corporate sector practices, notably labour and employment as well as supply chain and waste practices. Research examining strategies to counter harmful corporate practices [138] and associated tactics [24] may offer actionable insights beyond descriptions of the status quo. The German researchers already part of the international CDOH community can play an important role in supporting efforts to strengthen such research in the country.

For **research institutions**, a critical review of funding sources and the development of institutional COI strategies are warranted. Institutions can support CDOH researchers by providing structures that mitigate harms related to industry intimidation, e.g. by providing legal, technological, and psychosocial support. University lectures and workshops dedicated to the CDOH can raise the profile of this work among the next generation of researchers and practitioners.

**Research funders** can support local researchers in catching up with Anglo-Saxon research by issuing dedicated CDOH funding lines. Tendencies of both German and European funders to increasingly mandate health researchers to collaborate with industry should be examined critically.

## Supplementary Material

ckag030_Supplementary_Data

## Data Availability

Data extraction spreadsheets can be made available by the first authors upon reasonable request. Key points Research on the commercial determinants of health originating from Germany or including co-authors based in Germany is focused on the three unhealthy commodities ultra-processed food, tobacco, and alcohol, and on the pharmaceutical industry. The social network analysis of authorship networks shows largely disconnected clusters, indicating siloed work and little research across disciplines or industries. The term “commercial determinants” was only mentioned in 15 out of 136 included articles. Researchers and practitioners in Germany should embrace the concept of the commercial determinants of health to better address upstream causes of ill health.
