# Supplementary material for: Commercial determinants of health—a scoping review of research ‘made in Germany’
Source: Eur J Public Health. 2026 Mar 17;36(2):ckag030. doi: 10.1093/eurpub/ckag030 (PMC13017704; doi:10.1093/eurpub/ckag030)
Supplement: ckag030_Supplementary_Data [file ckag030_supplementary_data.zip › ejph-2025-04-om-0262-File008.pdf]

Additional file 4: Included articles

Study no	Publication (first author, year)	Title	DOI	Article type	Geographic focus	CDOH mentioned?
1	Achtyes, 2023	Telepsychiatry in an Era of Digital Mental Health Startups	10.1007/s11920-023-01425-9	overview	United States	no
2	Adams, 2011	Excise taxes on Tobacco and the problem of smuggling concerning the credibility of the Tobacco industry's "discarded-cigarette-packages- study"	10.1055/s-0030-1255089	other	Germany	no
3	Anderson, 2022	Lower Strength Alcohol Products—A Realist Review-Based Road Map for European Policy Making	10.3390/nu14183779	systematic review	unspecific	no
4	Babor, 2023	Alcohol: No Ordinary Commodity - A Summary of the Third Edition	10.1024/0939-5911/a000822	overview	unspecific	no
5	Barennes, 2016	Enforcing the International Code of Marketing of Breast-milk Substitutes for Better Promotion of Exclusive Breastfeeding	10.1177/0890334415607816	overview	LMICs	no
6	Batra, 2007	Funding support - Cui bono?	10.1111/j.1360-0443.2007.01891.x	opinion piece	Germany	no
7	Baur, 2019	How can the integrity of occupational and environmental health research be maintained in the presence of conflicting interests?	10.1186/s12940-019-0527-x	overview	unspecific	no
8	Baur, 2016	Asbestos: Socio-legal and Scientific Controversies and Unsound Science in the Context of the Worldwide Asbestos Tragedy - Lessons to be Learned	10.1055/s-0042-103580	opinion piece	unspecific	no
9	Baur, 2018	Asbestos-related disorders in Germany: Background, politics, incidence, diagnostics and compensation	10.3390/ijerph15010143	overview	Germany	no

10	Baur, 2015	Ethics, morality, and conflicting interests: how questionable professional integrity in some scientists supports global corporate influence in public health	10.1179/2049396714Y.0000000103	opinion piece	unspecific	no
11	Berner-Rodoreda, 2022	Commercial Influence on Political Declarations: The Crucial Distinction Between Consultation and Negotiation and the Need for Transparency in Lobbying Comment on "Competing Frames in Global Health Governance: An Analysis of Stakeholder Influence on the Political Declaration on Non-communicable Diseases"	10.34172/ijhpm.2021.132	opinion piece	UN member states	no
12	Bes-Rastrollo, 2013	Financial Conflicts of Interest and Reporting Bias Regarding the Association between Sugar-Sweetened Beverages and Weight Gain: A Systematic Review of Systematic Reviews	10.1371/journal.pmed.1001578	systematic review	unspecific	no
13	Boesen, 2021	Cross-sectional study of medical advertisements in a national general medical journal: evidence, cost, and safe use of advertised versus comparative drugs	10.1186/s41073-021-00111-9	primary - mixed	Denmark	no
14	Bognar, 2020	Promoting Breastfeeding and Interaction of Pediatric Associations With Providers of Nutritional Products	10.3389/fped.2020.562870	opinion piece	Europe	no
15	Burgmer, 2003	The "Sisi Syndrome": A new form of depression?	10.1007/s00115-003-1489-2	systematic review	Germany	no
16	Buzek, 2022	THE GLOBAL WEALTH CHAINS OF PRIVATE-EQUITY-RUN PHYSICIAN PRACTICES	10.1111/tesg.12519	primary - mixed	Germany	no
17	Cheung, 2023	Health and nutrition claims for infant formula: international cross sectional survey	10.1136/bmj-2022-071075	primary - mixed	unspecific	no
18	Coles, 2024	The Lancet Breast Cancer Commission	10.1016/S0140-6736(24)00747-5	overview	unspecific	yes

19	Deleye, 2014	Maternal health development programs: Comparing priorities of bilateral and private donors	10.1186/s12914-014-0031-x	primary - quantitative	LMICs	no
20	Dorlach, 2020	Interpreters of International Economic Law: Corporations and Bureaucrats in Contest over Chile's Nutrition Label	10.1111/lasr.12495	primary - qualitative	Chile	no
21	Dumbili, 2022	Making sense of ‘‘drink responsibly’’ messages: Explorations of the understanding and interpretations of young Nigerians who use alcohol	10.1016/j.drugpo.2022.103646	primary - qualitative	Nigeria	no
22	Dwivedi, 2023	Exploring the Darkverse: A Multi-Perspective Analysis of the Negative Societal Impacts of the Metaverse	10.1007/s10796-023-10400-x	opinion piece	unspecific	no
23	Effertz, 2022	Marketing of unhealthy food products to children	10.1007/s00112-021-01404-5	overview	Germany	no
24	Effertz, 2012	Do television food commercials target children in Germany?	10.1017/S1368980011003223	primary - mixed	Germany	no
25	Effertz, 2013	Cigarette prices, tobacco taxes and the proportion of contraband cigarettes in Germany	10.1055/s-0032-1321776	primary - quantitative	Germany	no
26	Erices, 2013	Criminal corruption of contracted medical practitioners and ethics Reflections on grey areas of corruption in the health care system	10.1007/s00481-012-0207-7	opinion piece	Germany	no
27	Faggion, 2014	Reporting of sources of funding in systematic reviews in periodontology and implant dentistry	10.1038/sj.bdj.2014.47	systematic review	unspecific	no
28	Fiedler, 2021	Corporate social responsibility vs. financial interests: the case of responsible gambling programs	10.1007/s10389-020-01219-w	primary - mixed	Germany	no
29	Fischer, 2014	Corruption in healthcare: a problem in Germany, too	10.20529/ijme.2014.028	opinion piece	Germany	no
30	Fleitmann, 2010	Women and smoking. A challenge for the tobacco control policy in Germany	10.1007/s00103-009-1005-3	other	Germany	no

31	Gadkaree, 2018	The role of industry influence in sinus balloon dilation: Trends over time	10.1002/lary.27203	primary - quantitative	United States	no
32	Gaudino, 2020	Characteristics of Contemporary Randomized Clinical Trials and Their Association with the Trial Funding Source in Invasive Cardiovascular Interventions	10.1001/jamainternmed.2020.1670	primary - quantitative	unspecific	no
33	Glaeske, 2007	The limits of economic efficiency - Medication provision and "border violations" through structures and interests	10.1016/j.zgesun.2007.04.012	overview	Germany	no
34	Gottlieb, 2020	The role of public-private partnerships in extending public healthcare provision to irregular migrants: stopgap or foot in the door?	10.1186/s13584-020-00406-0	opinion piece	Israel	no
35	Gray, 2022	Infant feeding policies and monitoring systems: A qualitativetative study of European Countries	10.1111/mcn.13425	primary - qualitative	Turkey, Ukraine, Croatia, Germany, Spain, Lithuania	no
36	Hanewinkel, 2004	Tobacco prevention programs financed by the tobacco industry are ineffective and only serve their interests	10.1024/suc.2004.50.2.134	opinion piece	Germany	no
37	Hartog, 2012	Systematic analysis of hydroxyethyl starch (HES) reviews: Proliferation of low-qualitativety reviews overwhelms the results of well-performed meta-analyses	10.1007/s00134-012-2614-0	systematic review	unspecific	no
38	Hauser, 2019	Medicinal cannabis and cannabis-based medication: an appeal to physicians, journalists, health insurances, and politicians for their responsible handling	10.1007/s00482-019-00409-0	overview	Germany	no
39	Heilmann, 2021	Reducing sugar consumption to improve oral health-which strategies are effective?	10.1007/s00103-021-03349-2	overview	Germany	yes
40	Heiss, 2021	Healthwashing in high-sugar food advertising: the effect of prior information on healthwashing perceptions in Austria	10.1093/heapro/daaa086	primary - quantitative	Austria	no
41	Hoe, 2022	Strategies to expand corporate autonomy by the tobacco, alcohol and sugar-	10.1186/s12992-022-00811-x	systematic review	unspecific	yes

		sweetened beverage industry: a scoping review of reviews				
42	Hoe, 2022	Understanding why collective action resulted in greater advances for tobacco control as compared to alcohol control during the Philippines' Sin Tax Reform: a qualitativetative study	10.1136/bmjopen-2021-054060	primary - qualitative	Philippines	no
43	Holst, 2023	The biomedical securitization of global health	10.1186/s12992-023-00915-y	overview	unspecific	yes
44	Huizinga, 2016	Food industry self-regulation scheme "EU Pledge" cannot prevent the marketing of unhealthy foods to children	10.1016/j.obmed.2016.01.004	primary - mixed	Germany	no
45	Jaeger, 2022	Describing and mapping scientific articles on alcohol globally for the period 2010-2021: a bibliometric analysis	10.1136/bmjopen-2022-063365	other	unspecific	yes
46	Jahnel, 2022	The digital rainbow: Digital determinants of health inequities	10.1177/20552076221129093	theoretical work	unspecific	yes
47	Jansen, 2019	Brucella-positive raw milk cheese sold on the inner European market: A public health threat due to illegal import?	10.1016/j.foodcont.2019.01.022	primary - quantitative	Germany	no
48	Jessani, 2022	Evidence attack in public health: Diverse actors' experiences with translating controversial or misrepresented evidence in health policy and systems research	10.1080/17441692.2021.2020319	primary - qualitative	unspecific	no
49	Jukola, 2021	Commercial interests, agenda setting, and the epistemic trustworthiness of nutrition science	10.1007/s11229-019-02228-3	theoretical work	unspecific	no
50	Kamal, 2015	Perceptions and attitudes of Egyptian health professionals and policy-makers towards pharmaceutical sales representatives and other promotional activities	10.1371/journal.pone.0140457	primary - qualitative	Egypt	no
51	Kickbusch, 2016	The commercial determinants of health	10.1016/S2214-109X%2816%2930217-0	theoretical work	unspecific	yes

52	Klemperer, 2008	Conflicts of interest: Danger for the doctor's judgment	NA	opinion piece	Germany	no
53	Knai, 2021	The case for developing a cohesive systems approach to research across unhealthy commodity industries	10.1136/bmjgh-2020-003543	primary - mixed	unspecific	no
54	Knai, 2018	Systems Thinking as a Framework for Analyzing Commercial Determinants of Health	10.1111/1468-0009.12339	theoretical work	unspecific	yes
55	Koch, 2020	Impact of physicians' participation in non-interventional post-marketing studies on their prescription habits: A retrospective 2-armed cohort study in Germany	10.1371/journal.pmed.1003151	primary - quantitative	Germany	no
56	Koletzko, 2014	Public-private collaboration in clinical research during pregnancy, lactation, and childhood: Joint position statement of the early nutrition academy and the European society for pediatric gastroenterology, hepatology, and nutrition	10.1097/MPG.0000000000000284	opinion piece	Europe	no
57	Krech, 2018	Banking for health: The role of financial sector actors in investing in global health	10.1136/bmjgh-2017-000597	other	unspecific	no
58	Kressing, 2018	Aspects of intercultural differences in the context of international medical tourism	10.1007/s00481-018-0498-4	other	unspecific	no
59	Küberling-Jost, 2021	Paths of Corporate Irresponsibility: A Dynamic Process	10.1007/s10551-019-04263-z	other	unspecific	no
60	Kyriss, 2008	The German Cigarette Industry Association - Obstructing effective tobacco control in Germany	10.1055/s-2008-1078752	primary - qualitative	Germany	no
61	Kyriss, 2013	The development of scientific consultants: How the tobacco industry creates controversy on the carcinogenicity of tobacco-specific nitrosamines	10.1136/tobaccocontrol-2012-050696	primary - qualitative	Germany	no
62	Landrigan, 2018	The Lancet Commission on pollution and health	10.1016/S0140-6736(17)32345-0	overview	unspecific	no

63	Landwehr, 2020	Industry self-regulation of food advertisement to children: Compliance versus effectiveness of the EU Pledge	10.1016/j.foodpol.2020.101833	primary - mixed	Germany	no
64	Lempert, 2018	Why German Neurology Needs an Annual Meeting Without Industry Sponsorship	10.1055/a-0584-5717	opinion piece	Germany	no
65	Leonardo Alves, 2017	Unbranded advertising of prescription medicines to the public by pharmaceutical companies	10.1002/14651858.CD012699	other	unspecific	no
66	Lieb, 2014	Contact between doctors and the pharmaceutical industry, their perceptions, and the effects on prescribing habits	10.1371/journal.pone.0110130	primary - quantitative	Germany	no
67	Lock, 2024	Argumentation strategies in lobbying: toward a typology	10.1108/JCOM-09-2022-0111	theoretical work	unspecific	no
68	Loss, 2009	Funding of community-based health promotion. The potentials of fundraising and public-private partnerships	10.1007/s11553-009-0175-z	overview	Germany	no
69	Loss, 2010	Direct-to-consumer (DTC) marketing of the pharmaceutical industry: Risks and ethical problems	10.1016/j.phf.2010.06.016	overview	unspecific	no
70	Ludwig, 2013	Gambling experiences, problems, research and policy: gambling in Germany	10.1111/j.1360-0443.2012.04025.x	overview	Germany	no
71	Meyer, 2023	Harm reduction in gambling: A public health task	10.1007/s11553-021-00924-y	overview	Germany	no
72	Mlinarić, 2020	Transnational tobacco companies and the mechanism of externalization: A realist synthesis	10.1016/j.healthplace.2019.102240	primary - qualitative	LMICs	no
73	Moerschel, 2022	Concepts of responsibility in the German media debate on sugar taxation: a qualitative framing analysis	10.1093/eurpub/ckab200	primary - qualitative	Germany, Mexico, UK	no
74	Moerschel, 2023	Evidence-related framing in the German debate on sugar taxation: a qualitative framing analysis and international comparison	10.1332/174426421X16448353303856	primary - qualitative	Germany, Mexico, United States, Chile, UK	no
75	Mons, 2010	Tobacco control politics in Germany. Evidence, success, and barriers	10.1007/s00103-009-1015-1	overview	Germany	no

76	Morgenstern, 2015	Content Themes of Alcohol Advertising in U.S. Television-Latent Class Analysis	10.1111/acer.12811	primary - mixed	United States	no
77	Morojele, 2021	Alcohol consumption, harms and policy developments in sub-Saharan Africa: The case for stronger national and regional responses	10.1111/dar.13247	overview	Sub-saharian Africa	no
78	Neufeld, 2020	Implementing health warnings on alcoholic beverages: On the leading role of countries of the commonwealth of independent states	10.3390/ijerph17218205	primary - qualitative	Commonwealth of Independent States (Armenia, Azerbaijan, Belarus, Kazakhstan, Kyrgyzstan, Moldova, Russia, Tajikistan, Turkmenistan, Uzbekistan)	no
79	Niebling, 2011	Bringing evidence to practice: Obstacles and barriers	10.1016/j.zefq.2011.10.027	overview	unspecific	no
80	Nordhagen, 2023	How do food companies try to reach lower-income consumers, and do they succeed? Insights from a systematic review	10.1016/j.gfs.2023.100699	systematic review	LMICs	no
81	Nordhausen, 2015	Nursing and industry relations: literature review and conflicts of interest survey	10.1016/j.zefq.2015.06.004	primary - mixed	unspecific	no
82	Nury, 2020	Impact of investigator initiated trials and industry sponsored trials on medical practice (IMPACT): Rationale and study design	10.1186/s12874-020-01125-5	other	unspecific	no
83	Odeigah, 2021	Alcohol Labeling Regulations and Industry Compliance in Nigeria: Evidence to Guide Policy Implementation	10.15288/jsad.2021.82.60	primary - mixed	Nigeria	no
84	Pattamatta, 2024	The value-for money of preventing and managing periodontitis: Opportunities and challenges	10.1111/prd.12569	overview	unspecific	yes

85	Peres, 2019	Oral diseases: a global public health challenge	10.1016/S0140-6736(19)31146-8	overview	unspecific	yes
86	Petticrew, 2017	'Nothing can be done until everything is done': the use of complexity arguments by food, beverage, alcohol and gambling industries	10.1136/jech-2017-209710	primary - qualitative	unspecific	no
87	Pieper, 2022	Impact of industry sponsorship on the qualitativety of systematic reviews of vaccines: a cross-sectional analysis of studies published from 2016 to 2019	10.1186/s13643-022-02051-x	other	unspecific	no
88	Popova, 2019	Alcohol industry-funded websites contribute to ambiguity regarding the harmful effects of alcohol consumption during pregnancy: A commentary on Lim et al. (2019)	10.15288/jsad.2019.80.534	opinion piece	unspecific	no
89	Probst, 2014	Association of industry sponsorship and positive outcome in randomised controlled trials in general and abdominal surgery: Protocol for a systematic review and empirical study	10.1186/2046-4053-3-138	other	unspecific	no
90	Probst, 2020	Conflicts of interest in randomised controlled surgical trials: Systematic review and qualitativetative and quantitativetative analysis	10.1515/iss-2016-0001	systematic review	unspecific	no
91	Probst, 2016	Industry Bias in Randomized Controlled Trials in General and Abdominal Surgery An Empirical Study	10.1097/SLA.0000000000001372	systematic review	unspecific	no
92	Przyrembel, 2012	Health claims on foods for children. Foods targeted at children	10.1007/s00112-012-2635-3	overview	EU member states	no
93	Robinson, 2020	Achieving a high level of protection from pesticides in Europe: Problems with the current risk assessment procedure and solutions	10.1017/err.2020.18	other	EU member states	no
94	Sahm, 2013	Of mugs, meals and more: The intricate relations between physicians and the medical industry	10.1007/s11019-012-9391-y	overview	unspecific	no

95	Schäffer, 2023	Conflicts of Interest in the Assessment of Chemicals, Waste, and Pollution	10.1021/acs.est.3c04213	overview	unspecific	no
96	Schaller, 2018	Tax on sugar sweetened beverages and influence of the industry to prevent regulation	10.4455/eu.2018.007	overview	unspecific	no
97	Schonhofer, 2004	Controlling corruption in order to improve global health	na	overview	unspecific	no
98	Schott, 2017	The influence of pharmaceutical companies on drug trials	10.1515/pubhef-2017-0018	overview	unspecific	no
99	Schott, 2010	The relation between publication bias and clinical trials funding	10.1016/j.zefq.2010.03.029	overview	unspecific	no
100	Schott, 2013	Does the Pharmaceutical Industry Influence Guidelines?	10.3238/arztebl.2013.0575	overview	Germany	no
101	Schrenk, 2021	Advertising or information in ophthalmology? : Scientific evaluation of a YouTube sample	10.1007/s00347-020-01105-6	primary - mixed	Germany	no
102	Singh, 2024	Reflections from COP28: Resisting healthwashing in climate change negotiations	10.1371/journal.pgph.0003076	opinion piece	unspecific	yes
103	Smith, 2016	Tobacco, Alcohol, and Processed Food Industries – Why Do Public Health Practitioners View Them So Differently?	10.3389/fpubh.2016.00064	opinion piece	unspecific	no
104	Sonntag, 2015	Beyond food promotion: A systematic review on the influence of the food industry on obesity-related dietary behaviour among children	10.3390/nu7105414	systematic review	European countries, United States, Canada, Australia, New Zealand	no
105	Spelsberg, 2017	Contribution of industry funded post-marketing studies to drug safety: Survey of notifications submitted to regulatory agencies	10.1136/bmj.j337	primary - mixed	Germany	no
106	Stein, 2022	The alcohol industry's involvement with road safety NGOs	10.1186/s12992-022-00813-9	primary - qualitative	unspecific	no
107	Stoll, 2020	Voluntary disclosures of payments from pharmaceutical companies to healthcare	10.1136/bmjopen-2020-037395	primary - quantitative	Germany	no

		professionals in Germany: A descriptive study of disclosures in 2015 and 2016				
108	Sun, 2011	The influence of study characteristics on reporting of subgroup analyses in randomised controlled trials: Systematic review	10.1136/bmj.d1569	systematic review	unspecific	no
109	Tan, 2013	Tobacco Company Efforts to Influence the Food and Drug Administration-Commissioned Institute of Medicine Report Clearing the Smoke: An Analysis of Documents Released through Litigation	10.1371/journal.pmed.1001450	primary - qualitative	United States	no
110	Theurich, 2018	Perspective: Novel Commercial Packaging and Devices for Complementary Feeding	10.1093/advances/nmy034	opinion piece	unspecific	no
111	Theurich, 2022	Moving Complementary Feeding Forward: Report on a Workshop of the Federation of International Societies for Pediatric Gastroenterology, Hepatology and Nutrition (FISPGHAN) and the World Health Organization Regional Office for Europe	10.1097/MPG.0000000000003562	other	unspecific	no
112	Theurich, 2024	National survey of infant feeding bottles in Germany: Their characteristics and marketing claims	10.1111/mcn.13632	primary - mixed	Germany	yes
113	Timotijevic, 2019	Research priority setting in food and health domain: European stakeholder beliefs about legitimacy criteria and processes	10.1016/j.foodpol.2018.12.005	primary - mixed	European Union	no
114	Töller, 2017	Voluntary Regulation by the Pharmaceutical Industry—Which Role for the Shadow of Hierarchy and Social Pressure?	10.1002/epa2.1006	theoretical work	Germany	no
115	Vandevijvere, 2023	Upstream Determinants of Overweight and Obesity in Europe	10.1007/s13679-023-00524-1	overview	Europe	yes
116	Von Philipsborn, 2018	Voluntary industry initiatives to promote healthy diets: A case study on a major European food retailer	10.1017/S1368980018002744	primary - qualitative	Germany	no
117	Watkins, 2018	Alma-Ata at 40 years: reflections from the Lancet Commission on Investing in Health	10.1016/S0140-6736(18)32389-4	overview	unspecific	yes

118	Watt, 2019	Ending the neglect of global oral health: time for radical action	10.1016/S0140-6736%2819%2931133-X	overview	unspecific	yes
119	Weiger, 2022	Seven-year tobacco tax plan in Ukraine: a case study of the actors, tactics and factors motivating policy passage	10.1136/bmjopen-2021-049833	primary - qualitative	Ukraine	no
120	Weishaar, 2016	Why media representations of corporations matter for public health policy: a scoping review	10.1186/s12889-016-3594-8	systematic review	unspecific	no
121	Wertz, 2011	The toxic effects of cigarette additives. Philip Morris' project mix reconsidered: An analysis of documents released through litigation	10.1371/journal.pmed.1001145	primary - qualitative	unspecific	no
122	Whitton, 2024	Ecological momentary assessment of digital food and beverage marketing exposure and impact in young adults: A feasibility study	10.1016/j.appet.2024.107338	primary - quantitative	Signapore	no
123	Wieseler, 2010	Finding studies on reboxetine: A tale of hide and seek	10.1136/bmj.c4942	overview	Germany	no
124	Wilhelm, 2007	Influence of industrial sources on children's health - Hot spot studies in North Rhine Westphalia, Germany	10.1016/j.ijheh.2007.02.007	primary - quantitative	Germany	no
125	Winkler, 2023	Patient data for commercial companies? An ethical framework for sharing patients' data with for-profit companies for research	10.1136/jme-2022-108781	overview	Europe	no
126	Zargaran, 2023	Cosmetic business mechanics in London: A cross-sectional analysis and audit of ASA compliance	10.1111/jocd.15750	primary - mixed	UK	no
**Search Update:**						
127	Bunder, 2021	Governing industry involvement in the non-communicable disease response in Kenya	10.1186/s12992-021-00776-3	primary - qualitative	Kenya	no
128	Erices, 2018	The role of the State Security Service (Stasi) in the context of international clinical	10.1371/journal.pone.0195017	primary - qualitative	Germany	no

		trials conducted by western pharmaceutical companies in Eastern Germany (1961-1990)				
129	Glaeske, 2008	Why are some drugs so expensive? The price policy of pharmaceutical companies - "Digging the grave of our health insurance-system"?	10.1016/j.zefq.2008.04.008	overview	Germany	no
130	Griefahn, 1997	Working in moderate cold: A possible risk to health	10.1539/joh.39.36	primary - quantitative	Germany	no
131	Herrera, 2016	Proximity to mining industry and respiratory diseases in children in a community in Northern Chile: A cross-sectional study	10.1186/s12940-016-0149-5	primary - quantitative	Chile	no
132	Khan, 2022	How conflicts of interest hinder effective regulation of healthcare: an analysis of antimicrobial use regulation in Cambodia, Indonesia and Pakistan	10.1136/bmjgh-2022-008596	primary - qualitative	Pakistan, Cambodia, Indonesia	no
133	Lerner, 2015	Possible association between celiac disease and bacterial transglutaminase in food processing: a hypothesis	10.1093/NUTRIT/NUV011	other	unspecific	no
134	Mechler, 2020	Disease awareness or subtle product placement? Orphan diseases featured in the television series "House, M.D." - a cross-sectional analysis	10.1186/s12910-020-0463-x	primary - qualitative	unspecific	no
135	Michaeli, 2024	Special FDA designations for drug development: orphan, fast track, accelerated approval, priority review, and breakthrough therapy	10.1007/s10198-023-01639-x	overview	United States	no
136	Trabert, 2024	Critical analysis of ginkgo preparations: comparison of approved drugs and dietary supplements marketed in Germany	10.1007/s00210-023-02602-6	primary - quantitative	Germany	no
