# Supplementary material for: Commercial determinants of health—a scoping review of research ‘made in Germany’
Source: Eur J Public Health. 2026 Mar 17;36(2):ckag030. doi: 10.1093/eurpub/ckag030 (PMC13017704; doi:10.1093/eurpub/ckag030)
Supplement: ckag030_Supplementary_Data [file ckag030_supplementary_data.zip › ejph-2025-04-om-0262-File005.pdf]

References

[continued from manuscript]

41. Paichadze N, Werbick M, Ndebele P, Bari I, Hyder AA. Commercial determinants of health: a proposed research agenda. Int J Public Health. 2020 Sep 1;65(7):1147–9.

42. Petticrew M, Glover RE, Volmink J, Blanchard L, Cott É, Knai C, et al. The Commercial Determinants of Health and Evidence Synthesis (CODES): methodological guidance for systematic reviews and other evidence syntheses. Syst Rev. 2023 Sep 14;12(1):165.

43. McKee M, Stuckler D. Revisiting the corporate and commercial determinants of health. Am J Public Health. 2018 Sep;108(9):1167–70.

44. Jasilionis D, van Raalte AA, Klüsener S, Grigoriev P. The underwhelming German life expectancy. Eur J Epidemiol. 2023 Aug 1;38(8):839–50.

45. Zeeb H, Loss J, Starke D, Altgeld T, Moebus S, Geffert K, et al. Public health in Germany: structures, dynamics, and ways forward. Lancet Public Health. 2025 Apr 1;10(4):e333–42.

46. AOK-Bundesverband, Deutsches Krebsforschungszentrum (DKFZ), editors. Public Health Index 2025. Gesundheitsschutz im europäischen Vergleich [Internet]. [cited 2026 Jan 19]. Available from: https://www.aok.de/pp/public-health/index

47. Eschmann A, Lange T. Lobbyreport 2024 [Internet]. Köln: LobbyControl – Initiative für Transparenz und Demokratie e.V.; 2024 Mar [cited 2026 Jan 19]. Available from: https://www.lobbycontrol.de/wp-content/uploads/lobbyreport-2024-web.pdf

48. Sell K, Nigg S, Leibinger A, Voss S, Klinger C, Rehfuess EA. ComDetGermany. 2024 Jul 29 [cited 2024 Nov 6]; Available from: https://osf.io/6pxzt/

49. Tricco AC, Lillie E, Zarin W, O'Brien KK, Colquhoun H, Levac D, et al. PRISMA Extension for Scoping Reviews (PRISMA-ScR): Checklist and Explanation. Ann Intern Med. 2018 Oct 2;169(7):467–73.

50. Dahlgren G, Whitehead M. Policies and strategies to promote social equity in health.

51. Bakkalbasi N, Bauer K, Glover J, Wang L. Three options for citation tracking: Google Scholar, Scopus and Web of Science. Biomed Digit Libr. 2006 Dec;3(1):7.

52. Hirt J, Nordhausen T, Fuerst T, Ewald H, Appenzeller-Herzog C. Guidance on terminology, application, and reporting of citation searching: the TARCiS statement. BMJ. 2024 May 9;385:e078384.

53. Vanhecke TE. Zotero. J Med Libr Assoc JMLA. 2008 Jul;96(3):275–6.

54. Ouzzani M, Hammady H, Fedorowicz Z, Elmagarmid A. Rayyan—a web and mobile app for systematic reviews. Syst Rev. 2016 Dec 5;5(1):210.

55. Schreier M. Qualitative content analysis in practice. Los Angeles: SAGE; 2012. 272 p.

56. Miake-Lye IM, Hempel S, Shanman R, Shekelle PG. What is an evidence map? A systematic review of published evidence maps and their definitions, methods, and products. Syst Rev. 2016 Dec;5(1):28.

57. Cowhitt T, Butler T, Wilson E. Using social network analysis to complete literature reviews: a new systematic approach for independent researchers to detect and interpret prominent research programs within large collections of relevant literature. Int J Soc Res Methodol. 2020 Sep 2;23(5):483–96.

58. Van Eck NJ, Waltman L. Software survey: VOSviewer, a computer program for bibliometric mapping. Scientometrics. 2010 Aug;84(2):523–38.

59. von Philipsborn P, Stratil JM, Heise TL, Landgraf R, Hauner H, Rehfuess EA. Voluntary industry initiatives to promote healthy diets: A case study on a major European food retailer. Public Health Nutr. 2018;21(18):3469–76.

60. Theurich MA, Ziebart M, Strobl F. National survey of infant feeding bottles in Germany: Their characteristics and marketing claims. Matern Child Nutr. 2024;20(3):e13632.

61. Burgmer M, Driesch G, Heuft G. The 'Sisi Syndrome': A new form of depression? Nervenarzt. 2003;74(5):440–4.

62. Mechler K, Rausch J, Mountford W, Ries M. Disease awareness or subtle product placement? Orphan diseases featured in the television series 'House, MD'-a cross-sectional analysis. BMC Med ETHICS. 2020 Mar 14;21(1).

63. Adams M, Effertz T. Excise taxes on Tobacco and the problem of smuggling concerning the credibility of the Tobacco industry's 'discarded-cigarette-packages- study'. Gesundheitswesen. 2011;73(10):705–12.

64. Weiger C, Hoe C, Cohen JE. Seven-year tobacco tax plan in Ukraine: a case study of the actors, tactics and factors motivating policy passage. BMJ Open. 2022 Feb 11;12(2):e049833.

65. Effertz T. Marketing of unhealthy food products to children. Monatsschr Kinderheilkd. 2022;170(2):133–8.

66. Probst P, Grummich K, Klaiber U, Knebel P, Ulrich A, Büchler MW, et al. Conflicts of interest in randomised controlled surgical trials: Systematic review and qualitative and quantitative analysis. Innov Surg Sci. 2020;1(1):33–9.

67. Wertz MS, Kyriss T, Paranjape S, Glantz SA. The toxic effects of cigarette additives. Philip Morris' project mix reconsidered: an analysis of documents released through litigation. PLoS Med. 2011 Dec;8(12):e1001145.

68. Schaller K, Mons U. Tax on sugar sweetened beverages and influence of the industry to prevent regulation. Ernahrungs Umsch. 2018;65(2):M82–9.

69. Stoll M, Hubenschmid L, Koch C, Lieb K. Voluntary disclosures of payments from pharmaceutical companies to healthcare professionals in Germany: a descriptive study of disclosures in 2015 and 2016. BMJ Open. 2020 Sep 1;10(9):e037395.

70. Kyriss T, Pötschke-Langer M, Grüning T. The German Cigarette Industry Association - Obstructing effective tobacco control in Germany. Gesundheitswesen. 2008;70(5):315–24.

71. Singh A, Ezzine T, Guinto RR, Gepp S, Parks RM, Thondoo M, et al. Reflections from COP28: Resisting healthwashing in climate change negotiations. PLOS Glob Public Health. 2024 Mar 28;4(3):e0003076.

72. Bognar Z, De Luca D, Domellof M, Hadjipanayis A, Haffner D, M. J, et al. Promoting Breastfeeding and Interaction of Pediatric Associations With Providers of Nutritional Products. Front Pediatr. 2020;8:562870.

73. Erices R, Frewer A, Gumz A. Criminal corruption of contracted medical practitioners and ethics Reflections on grey areas of corruption in the health care system. ETHIK Med. 2013;25(2):103–13.

74. Fischer C. Corruption in healthcare: a problem in Germany, too. Indian J Med Ethics. 2014;11(2):110–7.

75. Glaeske G. The limits of economic efficiency - Medication provision and ‘border violations’ through structures and interests. Z Arztl Fortbild Qualitatssich. 2007;101(5):347–55.

76. Kamal S, Holmberg C, Russell J, Bochenek T, Tobiasz-Adamczyk B, Fischer C, et al. Perceptions and Attitudes of Egyptian Health Professionals and Policy-Makers towards Pharmaceutical Sales Representatives and Other Promotional Activities. PLOS ONE. 2015 Oct 16;10(10):e0140457.

77. Klemperer D. Conflicts of interest: Danger for the doctor’s judgment. Dtsch Arzteblatt. 2008;105(40):A2098–100.

78. Koch C, Schleeff J, Techen F, Wollschläger D, Schott G, Kölbel R, et al. Impact of physicians’ participation in non-interventional post-marketing studies on their prescription habits: A retrospective 2-armed cohort study in Germany. PLOS Med. 2020 Jun 26;17(6):e1003151.

79. Lempert T, Janzen RWC, Diehl RR, Stark E, Paul F, Wilke C, et al. Why German Neurology Needs an Annual Meeting Without Industry Sponsorship. Aktuelle Neurol. 2018;45(6):429–33.

80. Niebling W. Bringing evidence to practice: Obstacles and barriers. Z Evidenz Fortbild Qual Im Gesundheitswesen. 2011;105(9):646–51.

81. Nordhausen T, Lins S, Panfil EM, Köpke S, Leino-Kilpi H, Langer G, et al. Nursing and industry relations: literature review and conflicts of interest survey. Z EVIDENZ Fortbild Qual IM GESUNDHEITSWESEN. 2015;109(8):621–31.

82. Sahm S. Of mugs, meals and more: The intricate relations between physicians and the medical industry. Med Health Care Philos. 2013;16(2):265–73.

83. Schonhofer PS. Controlling corruption in order to improve global health. Int J Risk Saf Med. 2004;16(3):195–205.

84. Holst J, van de Pas R. The biomedical securitization of global health. Glob Health. 2023 Mar 4;19(1):15.

85. Theurich MA, Fewtrell M, Baumgartner J, Perkin MR, Breda J, Wickramansinghe K, et al. Moving Complementary Feeding Forward: Report on a Workshop of the Federation of International Societies for Pediatric Gastroenterology, Hepatology and Nutrition (FISPGHAN) and the World Health Organization Regional Office for Europe. J Pediatr Gastroenterol Nutr. 2022;75(4):411–7.

86. Anderson P, Kokole D, Llopis EJ, Burton R, Lachenmeier DW. Lower Strength Alcohol Products—A Realist Review-Based Road Map for European Policy Making. Nutrients [Internet]. 2022 Sep 13;14(18). Available from: https://pubmed.ncbi.nlm.nih.gov/36145155/

87. Nordhagen S, Demmler KM. How do food companies try to reach lower-income consumers, and do they succeed? Insights from a systematic review. Glob Food Secur. 2023 Jun 1;37:100699.

88. Sonntag D, Schneider S, Mdege N, Ali S, Schmidt B. Beyond food promotion: A systematic review on the influence of the food industry on obesity-related dietary behaviour among children. Nutrients. 2015;7(10):8565–76.

89. Landrigan PJ, Fuller R, Acosta NJR, Adeyi O, Arnold R, Basu NN, et al. The Lancet Commission on pollution and health. The Lancet. 2018;391(10119):462–512.

90. Vandevijvere S, De Pauw R, Djojosoeparto S, Gorasso V, Guariguata L, Lovhaug AL, et al. Upstream Determinants of Overweight and Obesity in Europe. Curr Obes Rep. 2023;12(4):417–28.

91. Wilhelm M, Eberwein G, Hölzer J, Gladtke D, Angerer J, Marczynski B, et al. Influence of industrial sources on children's health – Hot spot studies in North Rhine Westphalia, Germany. Int J Hyg Environ Health. 2007 Oct 31;210(5):591–9.

92. Mlinarić M, Schreuders M, Graen L, Lessenich S. Transnational tobacco companies and the mechanism of externalization: A realist synthesis. Health Place. 2020 Jan 1;61:102240.

93. Pattamatta M, Chapple I, Listl S. The value-for money of preventing and managing periodontitis: Opportunities and challenges. Periodontol 2000. 2024;

94. Watkins DA, Yamey G, Schäferhoff M, Adeyi O, Alleyne G, Alwan A, et al. Alma-Ata at 40 years: reflections from the Lancet Commission on Investing in Health. The Lancet. 2018;392(10156):1434–60.

95. Watt RG, Daly B, Allison P, Macpherson LMD, Venturelli R, Listl S, et al. Ending the neglect of global oral health: time for radical action. The Lancet. 2019;394(10194):261–72.

96. Coles CE, Earl H, Anderson BO, Barrios CH, Bienz M, Bliss JM, et al. The Lancet Breast Cancer Commission. The Lancet. 2024;403(10439):1895–950.

97. Heilmann A, Ziller S. [Reducing sugar consumption to improve oral health-which strategies are effective? Bundesgesundheitsblatt Gesundheitsforschung Gesundheitsschutz. 2021;64(7):838–46.

98. Hoe C, Weiger C, Minosa MKR, Alonso F, Koon AD, Cohen JE. Strategies to expand corporate autonomy by the tobacco, alcohol and sugar-sweetened beverage industry: a scoping review of reviews. Glob Health. 2022;18(1):17.

99. Jaeger L, Devi T, Barbazza E, Neufeld M, Franz C, Marten R, et al. Describing and mapping scientific articles on alcohol globally for the period 2010–2021: a bibliometric analysis. BMJ Open. 2022 Sep 1;12(9):e063365.

100. Jahnel T, Dassow HH, Gerhardus A, Schüz B. The digital rainbow: Digital determinants of health inequities. Digit Health. 2022 Oct 2;8:20552076221129093.

101. Peres MA, Macpherson LMD, Weyant RJ, Daly B, Venturelli R, Mathur MR, et al. Oral diseases: a global public health challenge. LANCET. 2019;394(10194):249–60.

102. Heiss R, Naderer B, Matthes J. Healthwashing in high-sugar food advertising: the effect of prior information on healthwashing perceptions in Austria. Health Promot Int. 2021 Aug 1;36(4):1029–38.

103. Effertz T, Wilcke AC. Do television food commercials target children in Germany? Public Health Nutr. 2012;15(8):1466–73.

104. Smith K, Dorfman L, Freudenberg N, Hawkins B, Hilton S, Razum O, et al. Tobacco, Alcohol, and Processed Food Industries – Why Do Public Health Practitioners View Them So Differently? Front Public Health [Internet]. 2016 Apr 11 [cited 2026 Feb 9];4. Available from: https://www.frontiersin.org/journals/public-health/articles/10.3389/fpubh.2016.00064/full

105. Whitton C, Wong YHM, Lau J, Chua XH, Muller AM, Tan CS, et al. Ecological momentary assessment of digital food and beverage marketing exposure and impact in young adults: A feasibility study. Appetite. 2024;197:107338.

106. Glaeske G. Why are some drugs so expensive? The price policy of pharmaceutical companies - ‘Digging the grave of our health insurance-system’? Z Evidenz Fortbild Qual Im Gesundheitswesen. 2008;102(4):269-EP-277.

107. Spelsberg A, Prugger C, Doshi P, Ostrowski K, Witte T, Hüsgen D, et al. Contribution of industry funded post-marketing studies to drug safety: survey of notifications submitted to regulatory agencies. BMJ. 2017 Feb 7;356:j337.

108. Lieb K, Scheurich A. Contact between doctors and the pharmaceutical industry, their perceptions, and the effects on prescribing habits. PLoS ONE. 2014;9(10):e110130.

109. Batra A. Funding support - Cui bono? Addiction. 2007;102(7):1035.

110. Baur X, Budnik LT, Ruff K, Egilman DS, Lemen RA, Soskolne CL. Ethics, morality, and conflicting interests: how questionable professional integrity in some scientists supports global corporate influence in public health. Int J Occup Environ Health. 2015;21(2):172–5.

111. Gaudino M, Hameed I, Rahouma M, Khan FM, Tam DY, Biondi-Zoccai G, et al. Characteristics of Contemporary Randomized Clinical Trials and Their Association with the Trial Funding Source in Invasive Cardiovascular Interventions. JAMA Intern Med. 2020;180(7):993–1001.

112. Koletzko B, Benninga MA, Godfrey KM, Hornnes PJ, Kolaček S, Koletzko S, et al. Public-private collaboration in clinical research during pregnancy, lactation, and childhood: Joint position statement of the early nutrition academy and the european society for pediatric gastroenterology, hepatology, and nutrition. J Pediatr Gastroenterol Nutr. 2014;58(4):525–30.

113. Nury E, Bischoff K, Wollmann K, Nitschke K, Lohner S, Schumacher M, et al. Impact of investigator initiated trials and industry sponsored trials on medical practice (IMPACT): rationale and study design. BMC Med Res Methodol. 2020 Oct 2;20(1):246.

114. Pieper D, Hellbrecht I, Zhao L, Baur C, Pick G, Schneider S, et al. Impact of industry sponsorship on the quality of systematic reviews of vaccines: a cross-sectional analysis of studies published from 2016 to 2019. Syst Rev. 2022 Aug 22;11(1):174.

115. Probst P., Grummich K., Ulrich A., Buchler M.W., Knebel P., Diener M.K. Association of industry sponsorship and positive outcome in randomised controlled trials in general and abdominal surgery: Protocol for a systematic review and empirical study. Syst Rev. 2014;3(1):138.

116. Probst P, Knebel P, Grummich K, Tenckhoff S, Ulrich A, Büchler MW, et al. Industry Bias in Randomized Controlled Trials in General and Abdominal Surgery An Empirical Study. Ann Surg. 2016;264(1):87–92.

117. Schäffer A, Groh KJ, Sigmund G, Azoulay D, Backhaus T, Bertram MG, et al. Conflicts of Interest in the Assessment of Chemicals, Waste, and Pollution. Environ Sci Technol. 2023;57(48):19066–77.

118. Schott G, Dünnweber C, Mühlbauer B, Niebling W, Pachl H, Ludwig WD. Does the Pharmaceutical Industry Influence Guidelines? Dtsch ARZTEBLATT Int. 2013;110(35):575-U59.

119. Sun X, Briel M, Busse JW, You JJ, Akl EA, Mejza F, et al. The influence of study characteristics on reporting of subgroup analyses in randomised controlled trials: Systematic review. BMJ. 2011;342(7800):748.

120. Burgess RC, Nyhan K, Dharia N, Freudenberg N, Ransome Y. Characteristics of commercial determinants of health research on corporate activities: A scoping review. PLOS ONE. 2024 Apr 26;19(4):e0300699.

121. Kyvik S, Reymert I. Research collaboration in groups and networks: differences across academic fields. Scientometrics. 2017 Nov 1;113(2):951–67.

122. Hamilton CA, Vacca R, Stacciarini JMR. The emergence of team science: Understanding the state of adoption research through social network analysis. Adopt Foster. 2017 Dec;41(4):369–90.

123. Collin J, Plotnikova E, Hill S. One unhealthy commodities industry? Understanding links across tobacco, alcohol and ultra-processed food manufacturers and their implications for tobacco control and the SDGS. Tob Induc Dis [Internet]. 2018 Mar 1 [cited 2024 Oct 8];16(1). Available from: https://www.tobaccoinduceddiseases.org/One-unhealthy-commodities-industry-Understanding-links-across-tobacco-alcohol-and,83806,0,2.html

124. Fabbri A, Gilmore AB. Industry Influence on Science: What Is Happening and What Can Be Done. In: Maani N, Petticrew M, Galea S, editors. The Commercial Determinants of Health. 1st edn New York: Oxford University Press; 2022.

125. Fooks G. The Institutionalization of Corporate Power Within Policy. In: Maani N, Petticrew M, Galea S, editors. The Commercial Determinants of Health. 1st edn New York: Oxford University Press; 2022. p. 164-C17.P82.

126. Lacy-Nichols J, Cullerton K. A proposal for systematic monitoring of the commercial determinants of health: a pilot study assessing the feasibility of monitoring lobbying and political donations in Australia. Glob Health. 2023 Jan 7;19(1):2.

127. Lauber K, Rippin H, Wickramasinghe K, Gilmore AB. Corporate political activity in the context of sugar-sweetened beverage tax policy in the WHO European Region. Eur J Public Health. 2022 Oct 3;32(5):786–93.

128. Lauber K, McGee D, Gilmore AB. Commercial use of evidence in public health policy: a critical assessment of food industry submissions to global-level consultations on non-communicable disease prevention. BMJ Glob Health. 2021 Aug;6(8):e006176.

129. van den Akker A, Fabbri A, Bertscher A, Gilmore AB, Knai C, Cavill N, et al. Industry influence on public health policy formulation in the UK: a complex systems approach. Health Promot Int. 2024 Dec 1;39(6):daae139.

130. Knai C, Petticrew M, Douglas N, Durand MA, Eastmure E, Nolte E, et al. The Public Health Responsibility Deal: Using a Systems-Level Analysis to Understand the Lack of Impact on Alcohol, Food, Physical Activity, and Workplace Health Sub-Systems. Int J Environ Res Public Health. 2018 Dec 17;15(12):2895.

131. Hutchinson J, Rippin H, Threapleton D, Jewell J, Kanamäe H, Salupuu K, et al. High sugar content of European commercial baby foods and proposed updates to existing recommendations. Matern Child Nutr. 2020 Aug 30;17(1):e13020.

132. Buzek R, Scheuplein C. The global wealth chains of private-equity-run physician practices. Tijdschr VOOR Econ EN Soc Geogr. 2022;113(4):331–47.

133. Hagenaars L.L., Maani N., Schmidt L.A. Is the commercial determinants conversation confined to the health sciences? Potentially, and that’s a problem. Glob Health. 2024;20(1):3.

134. Haynes AS, Derrick GE, Redman S, Hall WD, Gillespie JA, Chapman S, et al. Identifying Trustworthy Experts: How Do Policymakers Find and Assess Public Health Researchers Worth Consulting or Collaborating With? Dowdy DW, editor. PLoS ONE. 2012 Mar;7(3):e32665–e32665.

135. Evans-Reeves KA, Matthes BK, Chamberlain P, Paichadze N, Gilmore AB, Mialon M. Intimidation against advocates and researchers in the tobacco, alcohol and ultra-processed food spaces: a review. Health Promot Int. 2024 Dec 1;39(6):daae153.

136. Pitt H, Thomas S, McCarthy S, van Schalkwyk MCI, Petticrew M, Randle M, et al. Developing structures to support researchers studying health-harming industries. Health Promot Int. 2024 Dec 1;39(6):daae174.

137. Pachanov A, Münte C, Hirt J, Pieper D. Development and validation of a geographic search filter for MEDLINE (PubMed) to identify studies about Germany. Res Synth Methods [Internet]. [cited 2024 Oct 30];n/a(n/a). Available from: https://onlinelibrary.wiley.com/doi/abs/10.1002/jrsm.1763

138. Friel S, Collin J, Daube M, Depoux A, Freudenberg N, Gilmore AB, et al. Commercial determinants of health: future directions. The Lancet. 2023 Apr 8;401(10383):1229–40.
