# Supplementary material for: Commercial determinants of health—a scoping review of research ‘made in Germany’
Source: Eur J Public Health. 2026 Mar 17;36(2):ckag030. doi: 10.1093/eurpub/ckag030 (PMC13017704; doi:10.1093/eurpub/ckag030)
Supplement: ckag030_Supplementary_Data [file ckag030_supplementary_data.zip › ejph-2025-04-om-0262-File006.pdf]

LMU LUDWIG-MAXIMILIANS-UNIVERSITÄT MÜNCHEN

PSPH

Additional file 2: Search strategies

Content

Search narrative ... 1

Search strand 1: List of seminal literature ... 1

Forward citation searching of seminal literature ... 17

Search strand 2: Database searching ... 19

Databases ... 20

Medline and Embase via OVID ... 20

Web of Science Core Collection ... 21

Google Scholar ... 23

Search narrative

The scoping review consists of two strands – forward citation searching of seminal CDOH literature (strand 1) and database searches (strand 2). Strand 1 is intended to identify CDOH research covering the breadth of the field. Strand 2 is intended to identify CDOH research which explicitly uses the term 'commercial determinants (of health)' or addresses key concepts including corporate sector practices and associated keywords, linked to a framework for CDOH (Gilmore et al., 2023).

Search strand 1: List of seminal literature

Articles cited more than 50 times and identified in a recent book on the commercial determinants of health (Maani et al., 2022), from the Lancet Commission series on CDOH, targeted Google Scholar searches, and searches for 'seminal' authors (more details below the table).

For articles cited multiple times in Maani et al., we list the first chapter in which the article was mentioned (e.g. Chapter 8, see below)

After review of citations of the original list of 222 seminal articles in Scopus, 63 references were removed (8 not found, 55 with under 50 citations). Newer articles (i.e. those published after publication of Maani et al. 2022) were included despite having less than 50 references.

Hence, we conducted forward citation searching of 159 articles: 134 references from the CDOH book, 3 references from the Lancet series, 11 references from the Google Scholar search, and 11 references authored by seminal authors.

	Reference	Categorisation (n citations)	Source
1.	Adams, P. J. (2007). Assessing whether to receive funding support from tobacco, alcohol, gambling and other dangerous consumption industries. Addiction, 102(7), 1027–1033. https://doi.org/10.1111/j.1360-0443.2007.01829.x	50-99	Chapter 8
2.	Amos, A. (2000). From social taboo to ‘torch of freedom’: The marketing of cigarettes to women. Tobacco Control, 9(1), 3–8. https://doi.org/10.1136/tc.9.1.3	250+	Chapter 6
3.	Apollonio, D. E., & Bero, L. A. (2007). The Creation of Industry Front Groups: The Tobacco Industry and “Get Government Off Our Back”. American Journal of Public Health, 97(3), 419–427. https://doi.org/10.2105/AJPH.2005.081117	100-149	Chapter 8
4.	Asante Antwi, H., Zhou, L., Xu, X., & Mustafa, T. (2021). Beyond COVID-19 Pandemic: An Integrative Review of Global Health Crisis Influencing the Evolution and Practice of Corporate Social Responsibility. Healthcare, 9(4), 453. https://doi.org/10.3390/healthcare9040453	50-99	GS search
5.	Babor, T. F. (2009). Alcohol research and the alcoholic beverage industry: Issues, concerns and conflicts of interest. Addiction, 104(s1), 34–47. https://doi.org/10.1111/j.1360-0443.2008.02433.x	150-199	Chapter 8
6.	Babor, T. F., & Robaina, K. (2013). Public Health, Academic Medicine, and the Alcohol Industry’s Corporate Social Responsibility Activities. American Journal of Public Health, 103(2), 206–214. https://doi.org/10.2105/AJPH.2012.300847	200-249	Chapter 6
7.	Baker, P., & Friel, S. (2016). Food systems transformations, ultra-processed food markets and the nutrition transition in Asia. Globalization and Health, 12(1), 80. https://doi.org/10.1186/s12992-016-0223-3	250+	Chapter 15
8.	Baker, P., Kay, A., & Walls, H. (2014). Trade and investment liberalization and Asia’s noncommunicable disease epidemic: A synthesis of data and existing literature. Globalization and Health, 10(1), 66. https://doi.org/10.1186/s12992-014-0066-8	100-149	Chapter 28
9.	Baker, P., Machado, P., Santos, T., Sievert, K., Backholer, K., Hadjikakou, M., Russell, C., Huse, O., Bell, C., Scrinis, G., Worsley, A., Friel, S., & Lawrence, M. (2020). Ultra-processed foods and the nutrition transition: Global, regional and national trends, food systems transformations and political economy drivers. Obesity Reviews, 21(12), e13126. https://doi.org/10.1111/obr.13126	250+	Chapter 9
10.	Baker, P., Russ, K., Kang, M., Santos, T. M., Neves, P. A. R., Smith, J., Kingston, G., Mialon, M., Lawrence, M., Wood, B., Moodie, R., Clark, D., Sievert, K., Boatwright, M., & McCoy, D. (2021). Globalization, first-foods systems transformations and corporate power: A synthesis of literature and data on the market and political practices of the transnational baby food	50-99	seminal author

	industry. Globalization and Health, 17(1), 58. https://doi.org/10.1186/s12992-021-00708-1		
11.	Baker, P., Smith, J. P., Garde, A., Grummer-Strawn, L. M., Wood, B., Sen, G., Hastings, G., Pérez-Escamilla, R., Ling, C. Y., Rollins, N., & McCoy, D. (2023). The political economy of infant and young child feeding: Confronting corporate power, overcoming structural barriers, and accelerating progress. The Lancet, 401(10375), 503–524. https://doi.org/10.1016/S0140-6736(22)01933-X	50-99	seminal author
12.	Barlow, P., McKee, M., Basu, S., & Stuckler, D. (2017). The health impact of trade and investment agreements: A quantitative systematic review and network co-citation analysis. Globalization and Health, 13(1), 13. https://doi.org/10.1186/s12992-017-0240-x	100-149	Chapter 9
13.	Barlow, P., Van Schalkwyk, M. C., McKee, M., Labonté, R., & Stuckler, D. (2021). COVID-19 and the collapse of global trade: Building an effective public health response. The Lancet Planetary Health, 5(2), e102–e107. https://doi.org/10.1016/S2542-5196(20)30291-6	100-149	seminal author
14.	Barnes, D. E. (1998). Why Review Articles on the Health Effects of Passive Smoking Reach Different Conclusions. JAMA, 279(19), 1566. https://doi.org/10.1001/jama.279.19.1566	250+	Chapter 19
15.	Barnes, D. E., & Bero, L. A. (1996). Industry-Funded Research and Conflict of Interest: An Analysis of Research Sponsored by the Tobacco Industry Through the Center for Indoor Air Research. Journal of Health Politics, Policy and Law, 21(3), 515–542. https://doi.org/10.1215/03616878-21-3-515	200-249	Chapter 19
16.	Baum, F. E., Sanders, D. M., Fisher, M., Anaf, J., Freudenberg, N., Friel, S., Labonté, R., London, L., Monteiro, C., Scott-Samuel, A., & Sen, A. (2016). Assessing the health impact of transnational corporations: Its importance and a framework. Globalization and Health, 12(1), 27. https://doi.org/10.1186/s12992-016-0164-x	100-149	Chapter 2
17.	Bero, L. (2003). Implications of the Tobacco Industry Documents for Public Health and Policy. Annual Review of Public Health, 24(1), 267–288. https://doi.org/10.1146/annurev.publhealth.24.100901.140813	250+	Chapter 8
18.	Blouin, C., Chopra, M., & Van Der Hoeven, R. (2009). Trade and social determinants of health. The Lancet, 373(9662), 502–507. https://doi.org/10.1016/S0140-6736(08)61777-8	250+	Chapter 14
19.	Bond, L. (2009). Access to Confidential Alcohol Industry Documents: From 'Big Tobacco' to 'Big Booze'. Australasian Medical Journal, 1(3), 1–26. https://doi.org/10.4066/AMJ.2009.43	50-99	Chapter 32
20.	Brandt, A. M. (2012). Inventing Conflicts of Interest: A History of Tobacco Industry Tactics. American Journal of Public Health, 102(1), 63–71. https://doi.org/10.2105/AJPH.2011.300292	250+	Chapter 22

21.	Brownell, K. D., & Warner, K. E. (2009). The Perils of Ignoring History: Big Tobacco Played Dirty and Millions Died. How Similar Is Big Food? The Milbank Quarterly, 87(1), 259–294. https://doi.org/10.1111/j.1468-0009.2009.00555.x	250+	Chapter 26
22.	Buchanan, L., Kelly, B., Yeatman, H., & Kariippanon, K. (2018). The Effects of Digital Marketing of Unhealthy Commodities on Young People: A Systematic Review. Nutrients, 10(2), 148. https://doi.org/10.3390/nu10020148	150-199	Chapter 33
23.	Buse, K., & Harmer, A. M. (2007). Seven habits of highly effective global public–private health partnerships: Practice and potential. Social Science & Medicine, 64(2), 259–271. https://doi.org/10.1016/j.socscimed.2006.09.001	250+	Chapter 22
24.	Buse, K., Tanaka, S., & Hawkes, S. (2017). Healthy people and healthy profits? Elaborating a conceptual framework for governing the commercial determinants of non-communicable diseases and identifying options for reducing risk exposure. Globalization and Health, 13(1), 34. https://doi.org/10.1186/s12992-017-0255-3	100-149	Chapter 2
25.	Cai, Y., Jo, H., & Pan, C. (2012). Doing Well While Doing Bad? CSR in Controversial Industry Sectors. Journal of Business Ethics, 108(4), 467–480. https://doi.org/10.1007/s10551-011-1103-7	250+	Chapter 29
26.	Carah, N., & Brodmerkel, S. (2021). Alcohol Marketing in the Era of Digital Media Platforms. Journal of Studies on Alcohol and Drugs, 82(1), 18–27. https://doi.org/10.15288/jsad.2021.82.18	50-99	Chapter 33
27.	Casswell, S. (2013). Vested interests in addiction research and policy. Why do we not see the corporate interests of the alcohol industry as clearly as we see those of the tobacco industry? Addiction, 108(4), 680–685. https://doi.org/10.1111/add.12011	150-199	Chapter 27
28.	Chartres, N., Fabbri, A., & Bero, L. A. (2016). Association of Industry Sponsorship With Outcomes of Nutrition Studies: A Systematic Review and Meta-analysis. JAMA Internal Medicine, 176(12), 1769. https://doi.org/10.1001/jamainternmed.2016.6721	50-99	Chapter 19
29.	Clapp, J. (2021). The problem with growing corporate concentration and power in the global food system. Nature Food, 2(6), 404–408. https://doi.org/10.1038/s43016-021-00297-7	200-249	GS search
30.	Collin, J. (2012). Tobacco control, global health policy and development: Towards policy coherence in global governance. Tobacco Control, 21(2), 274–280. https://doi.org/10.1136/tobaccocontrol-2011-050418	100-149	Chapter 22
31.	Courtemanche, C., & Carden, A. (2011). Supersizing supercenters? The impact of Walmart Supercenters on body mass index and obesity. Journal of Urban Economics, 69(2), 165–181. https://doi.org/10.1016/j.jue.2010.09.005	200-249	Chapter 33

32.	De Lacy-Vawdon, C., & Livingstone, C. (2020). Defining the commercial determinants of health: A systematic review. BMC Public Health, 20(1), 1022. https://doi.org/10.1186/s12889-020-09126-1	100-149	Chapter 2
33.	Dorfman, L., Cheyne, A., Friedman, L. C., Wadud, A., & Gottlieb, M. (2012). Soda and Tobacco Industry Corporate Social Responsibility Campaigns: How Do They Compare? PLoS Medicine, 9(6), e1001241. https://doi.org/10.1371/journal.pmed.1001241	200-249	Chapter 2
34.	Dorfman, L., Wallack, L., & Woodruff, K. (2005). More Than a Message: Framing Public Health Advocacy to Change Corporate Practices. Health Education & Behavior, 32(3), 320–336. https://doi.org/10.1177/1090198105275046	250+	Chapter 27
35.	Drope, J. (2001). Tobacco industry efforts at discrediting scientific knowledge of environmental tobacco smoke: A review of internal industry documents. Journal of Epidemiology & Community Health, 55(8), 588–594. https://doi.org/10.1136/jech.55.8.588	150-199	Chapter 22
36.	Fabbri, A., Lai, A., Grundy, Q., & Bero, L. A. (2018). The Influence of Industry Sponsorship on the Research Agenda: A Scoping Review. American Journal of Public Health, 108(11), e9–e16. https://doi.org/10.2105/AJPH.2018.304677	200-249	Chapter 3
37.	Fallah Shayan, N., Mohabbati-Kalejahi, N., Alavi, S., & Zahed, M. A. (2022). Sustainable Development Goals (SDGs) as a Framework for Corporate Social Responsibility (CSR). Sustainability, 14(3), 1222. https://doi.org/10.3390/su14031222	250-299	GS search
38.	Fletcher-Brown, J., Turnbull, S., Viglia, G., Chen, T., & Pereira, V. (2021). Vulnerable consumer engagement: How corporate social media can facilitate the replenishment of depleted resources. International Journal of Research in Marketing, 38(2), 518–529. https://doi.org/10.1016/j.ijresmar.2020.06.002	50-99	GS search
39.	Fooks, G. J., Gilmore, A. B., Smith, K. E., Collin, J., Holden, C., & Lee, K. (2011). Corporate Social Responsibility and Access to Policy Élites: An Analysis of Tobacco Industry Documents. PLoS Medicine, 8(8), e1001076. https://doi.org/10.1371/journal.pmed.1001076	150-199	Chapter 11
40.	Fooks, G. J., Williams, S., Box, G., & Sacks, G. (2019). Corporations' use and misuse of evidence to influence health policy: A case study of sugar-sweetened beverage taxation. Globalization and Health, 15(1), 56. https://doi.org/10.1186/s12992-019-0495-5	50-99	Chapter 5
41.	Friedman, L. C., Cheyne, A., Givelber, D., Gottlieb, M. A., & Daynard, R. A. (2015). Tobacco Industry Use of Personal Responsibility Rhetoric in Public Relations and Litigation: Disguising Freedom to Blame as Freedom of Choice. American Journal of Public Health, 105(2), 250–260. https://doi.org/10.2105/AJPH.2014.302226	50-99	Chapter 13

42.	Friel, S., Collin, J., Daube, M., Depoux, A., Freudenberg, N., Gilmore, A. B., Johns, P., Laar, A., Marten, R., McKee, M., & Mialon, M. (2023). Commercial determinants of health: Future directions. The Lancet, 401(10383), 1229–1240. https://doi.org/10.1016/S0140-6736(23)00011-9	50-99	Lancet series
43.	Friel, S., Hattersley, L., & Townsend, R. (2015). Trade Policy and Public Health. Annual Review of Public Health, 36(1), 325–344. https://doi.org/10.1146/annurev-publhealth-031914-122739	100-149	Chapter 3
44.	Fuchs, D., Kalfagianni, A., & Havinga, T. (2011). Actors in private food governance: The legitimacy of retail standards and multistakeholder initiatives with civil society participation. Agriculture and Human Values, 28(3), 353–367. https://doi.org/10.1007/s10460-009-9236-3	250+	Chapter 21
45.	Gerritsen, S., Sing, F., Lin, K., Martino, F., Backholer, K., Culpin, A., & Mackay, S. (2021). The Timing, Nature and Extent of Social Media Marketing by Unhealthy Food and Drinks Brands During the COVID-19 Pandemic in New Zealand. Frontiers in Nutrition, 8, 645349. https://doi.org/10.3389/fnut.2021.645349	50-90	GS search
46.	Gilmore, A. B., Fabbri, A., Baum, F., Bertscher, A., Bondy, K., Chang, H.-J., Demaio, S., Erzse, A., Freudenberg, N., Friel, S., Hofman, K. J., Johns, P., Abdool Karim, S., Lacy-Nichols, J., De Carvalho, C. M. P., Marten, R., McKee, M., Petticrew, M., Robertson, L., … Thow, A. M. (2023). Defining and conceptualising the commercial determinants of health. The Lancet, 401(10383), 1194–1213. https://doi.org/10.1016/S0140-6736(23)00013-2	200-249	Lancet series
47.	Gilmore, A. B., Fooks, G., Drope, J., Bialous, S. A., & Jackson, R. R. (2015). Exposing and addressing tobacco industry conduct in low-income and middle-income countries. The Lancet, 385(9972), 1029–1043. https://doi.org/10.1016/S0140-6736(15)60312-9	250+	Chapter 11
48.	Gilmore, A. B., Savell, E., & Collin, J. (2011). Public health, corporations and the New Responsibility Deal: Promoting partnerships with vectors of disease? Journal of Public Health, 33(1), 2–4. https://doi.org/10.1093/pubmed/fdr008	150-199	Chapter 21
49.	Gilmore, A. B., Tavakoly, B., Taylor, G., & Reed, H. (2013). Understanding tobacco industry pricing strategy and whether it undermines tobacco tax policy: The example of the UK cigarette market. Addiction, 108(7), 1317–1326. https://doi.org/10.1111/add.12159	150-199	Chapter 11
50.	Glantz, S. A. (1995). Looking Through a Keyhole at the Tobacco Industry: The Brown and Williamson Documents. JAMA, 274(3), 219. https://doi.org/10.1001/jama.1995.03530030039032	150-199	Chapter 11

51.	Gleeson, D., & Friel, S. (2013). Emerging threats to public health from regional trade agreements. The Lancet, 381(9876), 1507–1509. https://doi.org/10.1016/S0140-6736(13)60312-8	100-149	Chapter 32
52.	Gøtzsche, P. C., Hróbjartsson, A., Johansen, H. K., Haahr, M. T., Altman, D. G., & Chan, A.-W. (2006). Constraints on Publication Rights in Industry-Initiated Clinical Trials. JAMA, 295(14), 1641. https://doi.org/10.1001/jama.295.14.1645	100-149	Chapter 8
53.	Grüning, T., Gilmore, A. B., & McKee, M. (2006). Tobacco Industry Influence on Science and Scientists in Germany. American Journal of Public Health, 96(1), 20–32. https://doi.org/10.2105/AJPH.2004.061507	150-199	Chapter 24
54.	Hahn, R. A., Middleton, J. C., Elder, R., Brewer, R., Fielding, J., Naimi, T. S., Toomey, T. L., Chattopadhyay, S., Lawrence, B., & Campbell, C. A. (2012). Effects of Alcohol Retail Privatization on Excessive Alcohol Consumption and Related Harms. American Journal of Preventive Medicine, 42(4), 418–427. https://doi.org/10.1016/j.amepre.2012.01.002	100-149	Chapter 10
55.	Hancock, L., & Smith, G. (2017). Critiquing the Reno Model I-IV International Influence on Regulators and Governments (2004–2015)—The Distorted Reality of “Responsible Gambling”. International Journal of Mental Health and Addiction, 15(6), 1151–1176. https://doi.org/10.1007/s11469-017-9746-y	150-199	Chapter 13
56.	Hansen, C., Lundh, A., Rasmussen, K., & Hróbjartsson, A. (2019). Financial conflicts of interest in systematic reviews: Associations with results, conclusions, and methodological quality. Cochrane Database of Systematic Reviews, 2019(8). https://doi.org/10.1002/14651858.MR000047.pub2	50-99	Chapter 19
57.	Hawkins, B., & Holden, C. (2013). Framing the alcohol policy debate: Industry actors and the regulation of the UK beverage alcohol market. Critical Policy Studies, 7(1), 53–71. https://doi.org/10.1080/19460171.2013.766023	100-149	Chapter 4
58.	Hawkins, B., & Holden, C. (2014). ‘Water dripping on stone’? Industry lobbying and UK alcohol policy. Policy & Politics, 42(1), 55–70. https://doi.org/10.1332/030557312X655468	100-149	Chapter 4
59.	Hawkins, B., & McCambridge, J. (2014). Industry Actors, Think Tanks, and Alcohol Policy in the United Kingdom. American Journal of Public Health, 104(8), 1363–1369. https://doi.org/10.2105/AJPH.2013.301858	50-99	Chapter 27
60.	Hawkins, B., Holden, C., Eckhardt, J., & Lee, K. (2018). Reassessing policy paradigms: A comparison of the global tobacco and alcohol industries. Global Public Health, 13(1), 1–19. https://doi.org/10.1080/17441692.2016.1161815	100-149	Chapter 2
61.	Hiscock, R., Branston, J. R., McNeill, A., Hitchman, S. C., Partos, T. R., & Gilmore, A. B. (2018). Tobacco industry strategies undermine government tax policy: Evidence from commercial data. Tobacco Control, 27(5), 488–497. https://doi.org/10.1136/tobaccocontrol-2017-053891	100-149	Chapter 11

62.	Holden, C., & Hawkins, B. (2013). 'Whisky gloss': The alcohol industry, devolution and policy communities in Scotland. Public Policy and Administration, 28(3), 253–273. https://doi.org/10.1177/0952076712452290	50-99	Chapter 4
63.	Hong, M.-K. (2002). How the tobacco industry responded to an influential study of the health effects of secondhand smoke. BMJ, 325(7377), 1413–1416. https://doi.org/10.1136/bmj.325.7377.1413	100-149	Chapter 8
64.	Huss, A., Egger, M., Hug, K., Huwiler-Müntener, K., & Röösli, M. (2007). Source of Funding and Results of Studies of Health Effects of Mobile Phone Use: Systematic Review of Experimental Studies. Environmental Health Perspectives, 115(1), 1–4. https://doi.org/10.1289/ehp.9149	250+	Chapter 19
65.	Ioannidis, J. P. A., & Trepanowski, J. F. (2018). Disclosures in Nutrition Research: Why It Is Different. JAMA, 319(6), 547. https://doi.org/10.1001/jama.2017.18571	50-99	Chapter 30
66.	Jahiel, R. I. (2008). Corporation-induced Diseases, Upstream Epidemiologic Surveillance, and Urban Health. Journal of Urban Health, 85(4), 517–531. https://doi.org/10.1007/s11524-008-9283-x	50-99	Chapter 21
67.	Jahiel, R. I., & Babor, T. F. (2007). Industrial epidemics, public health advocacy and the alcohol industry: Lessons from other fields. Addiction, 102(9), 1335–1339. https://doi.org/10.1111/j.1360-0443.2007.01900.x		Chapter 4
68.	Katikireddi, S. V., Bond, L., & Hilton, S. (2014). Changing Policy Framing as a Deliberate Strategy for Public Health Advocacy: A Qualitative Policy Case Study of Minimum Unit Pricing of Alcohol. The Milbank Quarterly, 92(2), 250–283. https://doi.org/10.1111/1468-0009.12057	50-99	Chapter 27
69.	Katz, D., Caplan, A. L., & Merz, J. F. (2010). All Gifts Large and Small: Toward an Understanding of the Ethics of Pharmaceutical Industry Gift-Giving. The American Journal of Bioethics, 10(10), 11–17. https://doi.org/10.1080/15265161.2010.519226	250+	Chapter 22
70.	Kearns, C. E., Glantz, S. A., & Schmidt, L. A. (2015). Sugar Industry Influence on the Scientific Agenda of the National Institute of Dental Research's 1971 National Caries Program: A Historical Analysis of Internal Documents. PLOS Medicine, 12(3), e1001798. https://doi.org/10.1371/journal.pmed.1001798	150-199	Chapter 8
71.	Kickbusch, I., Allen, L., & Franz, C. (2016). The commercial determinants of health. The Lancet Global Health, 4(12), e895–e896. https://doi.org/10.1016/S2214-109X(16)30217-0	250+	Chapter 2
72.	King, M., Essick, C., Bearman, P., & Ross, J. S. (2013). Medical school gift restriction policies and physician prescribing of newly marketed psychotropic medications: Difference-in-differences analysis. BMJ, 346(jan30 5), f264–f264. https://doi.org/10.1136/bmj.f264	100-149	Chapter 8

73.	Knai, C., Petticrew, M., Mays, N., Capewell, S., Cassidy, R., Cummins, S., Eastmure, E., Fafard, P., Hawkins, B., Jensen, J. D., Katikireddi, S. V., Mwatsama, M., Orford, J., & Weishaar, H. (2018). Systems Thinking as a Framework for Analyzing Commercial Determinants of Health. The Milbank Quarterly, 96(3), 472–498. https://doi.org/10.1111/1468-0009.12339	100-149	Chapter 2
74.	Kraak, V. I., Harrigan, P. B., Lawrence, M., Harrison, P. J., Jackson, M. A., & Swinburn, B. (2012). Balancing the benefits and risks of public–private partnerships to address the global double burden of malnutrition. Public Health Nutrition, 15(3), 503–517. https://doi.org/10.1017/S1368980011002060	150-199	Chapter 29
75.	Krumholz, H. M., Ross, J. S., Presler, A. H., & Egilman, D. S. (2007). What have we learnt from Vioxx? BMJ, 334(7585), 120–123. https://doi.org/10.1136/bmj.39024.487720.68	250+	Chapter 8
76.	Kunkel, D. L., Castonguay, J. S., & Filer, C. R. (2015). Evaluating Industry Self-Regulation of Food Marketing to Children. American Journal of Preventive Medicine, 49(2), 181–187. https://doi.org/10.1016/j.amepre.2015.01.027	100-149	Chapter 2
77.	Lacy-Nichols, J., Marten, R., Crosbie, E., & Moodie, R. (2022). The public health playbook: Ideas for challenging the corporate playbook. The Lancet Global Health, 10(7), e1067–e1072. https://doi.org/10.1016/S2214-109X(22)00185-1	50-99	GS search
78.	Lacy-Nichols, J., Nandi, S., Mialon, M., McCambridge, J., Lee, K., Jones, A., Gilmore, A. B., Galea, S., De Lacy-Vawdon, C., De Carvalho, C. M. P., Baum, F., & Moodie, R. (2023). Conceptualising commercial entities in public health: Beyond unhealthy commodities and transnational corporations. The Lancet, 401(10383), 1214–1228. https://doi.org/10.1016/S0140-6736(23)00012-0	50-99	Lancet series
79.	Landman, A., Ling, P. M., & Glantz, S. A. (2002). Tobacco Industry Youth Smoking Prevention Programs: Protecting the Industry and Hurting Tobacco Control. American Journal of Public Health, 92(6), 917–930. https://doi.org/10.2105/AJPH.92.6.917	250+	Chapter 6
80.	Lauber, K., Rutter, H., & Gilmore, A. B. (2021). Big food and the World Health Organization: A qualitative study of industry attempts to influence global-level non-communicable disease policy. BMJ Global Health, 6(6), e005216. https://doi.org/10.1136/bmjgh-2021-005216	50-99	seminal author
81.	Lesser, L. I., Ebbeling, C. B., Goozner, M., Wypij, D., & Ludwig, D. S. (2007). Relationship between Funding Source and Conclusion among Nutrition-Related Scientific Articles. PLoS Medicine, 4(1), e5. https://doi.org/10.1371/journal.pmed.0040005	250+	Chapter 19
82.	Lilienfeld, D. E. (1991). The silence: The asbestos industry and early occupational cancer research--a case study. American Journal of Public Health, 81(6), 791–800. https://doi.org/10.2105/AJPH.81.6.791	100-149	Chapter 8

83.	Lim, A. W. Y., Van Schalkwyk, M. C. I., Maani Hessari, N., & Petticrew, M. P. (2019). , Fertility, Breastfeeding, and Alcohol Consumption: An Analysis of Framing and Completeness of Information Disseminated by Alcohol Industry–Funded Organizations. Journal of Studies on Alcohol and Drugs, 80(5), 524–533. https://doi.org/10.15288/jsad.2019.80.524	50-99	Chapter 6
84.	Liu, W., Xu, Y., Fan, D., Li, Y., Shao, X.-F., & Zheng, J. (2021). Alleviating corporate environmental pollution threats toward public health and safety: The role of smart city and artificial intelligence. Safety Science, 143, 105433. https://doi.org/10.1016/j.ssci.2021.105433	50-99	GS search
85.	Lundh, A., Lexchin, J., Mintzes, B., Schroll, J. B., & Bero, L. (2017). Industry sponsorship and research outcome. Cochrane Database of Systematic Reviews, 2017(2). https://doi.org/10.1002/14651858.MR000033.pub3	250+	Chapter 8
86.	Maani Hessari, N., & Petticrew, M. (2018). What does the alcohol industry mean by 'Responsible drinking'? A comparative analysis. Journal of Public Health, 40(1), 90–97. https://doi.org/10.1093/pubmed/fdx040	50-99	Chapter 6
87.	Maani, N., Collin, J., Friel, S., Gilmore, A. B., McCambridge, J., Robertson, L., & Petticrew, M. P. (2020). Bringing the commercial determinants of health out of the shadows: A review of how the commercial determinants are represented in conceptual frameworks. European Journal of Public Health, 30(4), 660–664. https://doi.org/10.1093/eurpub/ckz197	100-149	Chapter 1
88.	Macassa, G., McGrath, C., Tomaselli, G., & Buttigieg, S. C. (2021). Corporate social responsibility and internal stakeholders' health and well-being in Europe: A systematic descriptive review. Health Promotion International, 36(3), 866–883. https://doi.org/10.1093/heapro/daaa071	50-99	GS search
89.	Madureira Lima, J., & Galea, S. (2018). Corporate practices and health: A framework and mechanisms. Globalization and Health, 14(1), 21. https://doi.org/10.1186/s12992-018-0336-y	100-149	Chapter 1
90.	Malone, R. E., Grundy, Q., & Bero, L. A. (2012). Tobacco industry denormalisation as a tobacco control intervention: A review. Tobacco Control, 21(2), 162–170. https://doi.org/10.1136/tobaccocontrol-2011-050200	150-199	Chapter 11
91.	Markham, F., & Young, M. (2015). "Big Gambling": The rise of the global industry-state gambling complex. Addiction Research & Theory, 23(1), 1–4. https://doi.org/10.3109/16066359.2014.929118	100-149	Chapter 13
92.	McCambridge, J., Mialon, M., & Hawkins, B. (2018). Alcohol industry involvement in policymaking: A systematic review. Addiction, 113(9), 1571–1584. https://doi.org/10.1111/add.14216	250+	Chapter 4
93.	McKee, M., & Stuckler, D. (2018). Revisiting the Corporate and Commercial Determinants of Health. American Journal of Public		Chapter 4

	Health, 108(9), 1167–1170. https://doi.org/10.2105/AJPH.2018.304510		
94.	Mialon, M. (2020). An overview of the commercial determinants of health. Globalization and Health, 16(1), 74. https://doi.org/10.1186/s12992-020-00607-x	200-249	Chapter 2
95.	Mialon, M., Gaitan Charry, D. A., Cediel, G., Crosbie, E., Scagliusi, F. B., & Perez Tamayo, E. M. (2021). 'I had never seen so many lobbyists': Food industry political practices during the development of a new nutrition front-of-pack labelling system in Colombia. Public Health Nutrition, 24(9), 2737–2745. https://doi.org/10.1017/S1368980020002268	50-99	Seminal author
96.	Mialon, M., Swinburn, B., & Sacks, G. (2015). A proposed approach to systematically identify and monitor the corporate political activity of the food industry with respect to public health using publicly available information. Obesity Reviews, 16(7), 519–530. https://doi.org/10.1111/obr.12289	250+	Chapter 2
97.	Mialon, M., Swinburn, B., Wate, J., Tukana, I., & Sacks, G. (2016). Analysis of the corporate political activity of major food industry actors in Fiji. Globalization and Health, 12(1), 18. https://doi.org/10.1186/s12992-016-0158-8	100-149	Chapter 29
98.	Mialon, M., Vandevijvere, S., Carriedo-Lutzenkirchen, A., Bero, L., Gomes, F., Petticrew, M., McKee, M., Stuckler, D., & Sacks, G. (2020). Mechanisms for addressing and managing the influence of corporations on public health policy, research and practice: A scoping review. BMJ Open, 10(7), e034082. https://doi.org/10.1136/bmjopen-2019-034082	50-99	Chapter 32
99.	Millar, J. S. (2013). The Corporate Determinants of Health: How Big Business Affects Our Health, and the Need for Government Action! Canadian Journal of Public Health, 104(4), e327–e329. https://doi.org/10.17269/cjph.104.3849	50-99	Chapter 21
100.	Miller, D., & Harkins, C. (2010). Corporate strategy, corporate capture: Food and alcohol industry lobbying and public health. Critical Social Policy, 30(4), 564–589. https://doi.org/10.1177/0261018310376805	200-249	Chapter 5
101.	Milsom, P., Smith, R., Baker, P., & Walls, H. (2021). Corporate power and the international trade regime preventing progressive policy action on non-communicable diseases: A realist review. Health Policy and Planning, 36(4), 493–508. https://doi.org/10.1093/heapol/czaa148	50-99	GS search
102.	Mishra, L. (2021). Corporate social responsibility and sustainable development goals: A study of Indian companies. Journal of Public Affairs, 21(1), e2147. https://doi.org/10.1002/pa.2147	50-99	GS search
103.	Monteiro, C. A., & Cannon, G. (2012). The Impact of Transnational "Big Food" Companies on the South: A View from Brazil. PLoS Medicine, 9(7), e1001252. https://doi.org/10.1371/journal.pmed.1001252	250+	Chapter 3

104.	Moodie, R., Bennett, E., Kwong, E. J. L., Santos, T. M., Pratiwi, L., Williams, J., & Baker, P. (2021). Ultra-Processed Profits: The Political Economy of Countering the Global Spread of Ultra-Processed Foods – A Synthesis Review on the Market and Political Practices of Transnational Food Corporations and Strategic Public Health Responses. International Journal of Health Policy and Management, 1. https://doi.org/10.34172/ijhpm.2021.45	100-149	seminal author
105.	Moodie, R., Stuckler, D., Monteiro, C., Sheron, N., Neal, B., Thamarangsi, T., Lincoln, P., & Casswell, S. (2013). Profits and pandemics: Prevention of harmful effects of tobacco, alcohol, and ultra-processed food and drink industries. The Lancet, 381(9867), 670–679. https://doi.org/10.1016/S0140-6736(12)62089-3	250+	Chapter 3
106.	Moynihan, R. (2008). Key opinion leaders: Independent experts or drug representatives in disguise? BMJ, 336(7658), 1402–1403. https://doi.org/10.1136/bmj.39575.675787.651	250+	Chapter 8
107.	Nestle, M. (2001). Food company sponsorship of nutrition research and professional activities: A conflict of interest? Public Health Nutrition, 4(5), 1015–1022. https://doi.org/10.1079/PHN2001253	100-149	Chapter 30
108.	Nixon, L., Mejia, P., Cheyne, A., Wilking, C., Dorfman, L., & Daynard, R. (2015). "We're Part of the Solution": Evolution of the Food and Beverage Industry's Framing of Obesity Concerns Between 2000 and 2012. American Journal of Public Health, 105(11), 2228–2236. https://doi.org/10.2105/AJPH.2015.302819	50-99	Chapter 2
109.	Ong, E. K., & Glantz, S. A. (2001). Constructing "Sound Science" and "Good Epidemiology": Tobacco, Lawyers, and Public Relations Firms. American Journal of Public Health, 91(11), 1749–1757. https://doi.org/10.2105/AJPH.91.11.1749	250+	Chapter 8
110.	Palazzo, G., & Richter, U. (2005). CSR Business as Usual? The Case of the Tobacco Industry. Journal of Business Ethics, 61(4), 387–401. https://doi.org/10.1007/s10551-005-7444-3	250+	Chapter 33
111.	Panjwani, C., & Caraher, M. (2014). The Public Health Responsibility Deal: Brokering a deal for public health, but on whose terms? Health Policy, 114(2–3), 163–173. https://doi.org/10.1016/j.healthpol.2013.11.002	100-149	Chapter 2
112.	Peeters, S., Costa, H., Stuckler, D., McKee, M., & Gilmore, A. B. (2015). The revision of the 2014 European tobacco products directive: An analysis of the tobacco industry's attempts to 'break the health silo'. Tobacco Control, tobaccocontrol-2014-051919. https://doi.org/10.1136/tobaccocontrol-2014-051919	50-99	Chapter 11
113.	Pérez-Escamilla, R., Tomori, C., Hernández-Cordero, S., Baker, P., Barros, A. J. D., Bégin, F.,chapman, D. J., Grummer-Strawn, L. M., McCoy, D., Menon, P., Ribeiro Neves, P. A., Piwoz, E., Rollins, N., Victora, C. G., & Richter, L. (2023). Breastfeeding:	200-249	seminal author

	Crucially important, but increasingly challenged in a market-driven world. The Lancet, 401(10375), 472–485. https://doi.org/10.1016/S0140-6736(22)01932-8		
114.	Petticrew, M., Katikireddi, S. V., Knai, C., Cassidy, R., Maani Hessari, N., Thomas, J., & Weishaar, H. (2017). 'Nothing can be done until everything is done': The use of complexity arguments by food, beverage, alcohol and gambling industries. Journal of Epidemiology and Community Health, 71(11), 1078–1083. https://doi.org/10.1136/jech-2017-209710	100-149	Chapter 6
115.	Petticrew, M., Maani Hessari, N., Knai, C., & Weiderpass, E. (2018). How alcohol industry organisations mislead the public about alcohol and cancer. Drug and Alcohol Review, 37(3), 293–303. https://doi.org/10.1111/dar.12596	250+	Chapter 2
116.	Petticrew, M., Maani, N., Pettigrew, L., Rutter, H., & Van Schalkwyk, M. C. (2020). Dark Nudges and Sludge in Big Alcohol: Behavioral Economics, Cognitive Biases, and Alcohol Industry Corporate Social Responsibility. The Milbank Quarterly, 98(4), 1290–1328. https://doi.org/10.1111/1468-0009.12475	100-149	Chapter 24
117.	Ravuvu, A., Friel, S., Thow, A.-M., Snowdon, W., & Wate, J. (2017). Monitoring the impact of trade agreements on national food environments: Trade imports and population nutrition risks in Fiji. Globalization and Health, 13(1), 33. https://doi.org/10.1186/s12992-017-0257-1	50-99	Chapter 14
118.	Reich, M. R. (2000). Public–private partnerships for public health. Nature Medicine, 6(6), 617–620. https://doi.org/10.1038/76176	250+	Chapter 31
119.	Rodwin, M. A. (2012). Conflicts of Interest, Institutional Corruption, and Pharma: An Agenda for Reform. Journal of Law, Medicine & Ethics, 40(3), 511–522. https://doi.org/10.1111/j.1748-720X.2012.00683.x	50-99	Chapter 22
120.	Rollins, N., Piwoz, E., Baker, P., Kingston, G., Mabaso, K. M., McCoy, D., Ribeiro Neves, P. A., Pérez-Escamilla, R., Richter, L., Russ, K., Sen, G., Tomori, C., Victora, C. G., Zambrano, P., & Hastings, G. (2023). Marketing of commercial milk formula: A system to capture parents, communities, science, and policy. The Lancet, 401(10375), 486–502. https://doi.org/10.1016/S0140-6736(22)01931-6	100-149	seminal author
121.	Ross, J. S., Hill, K. P., Egilman, D. S., & Krumholz, H. M. (2008). Guest Authorship and Ghostwriting in Publications Related to Rofecoxib: A Case Study of Industry Documents From Rofecoxib Litigation. JAMA, 299(15), 1800. https://doi.org/10.1001/jama.299.15.1800	250+	Chapter 8
122.	Ruckert, A., & Labonté, R. (2014). Public–private partnerships in global health: The good, the bad and the ugly. Third World Quarterly, 35(9), 1598–1614. https://doi.org/10.1080/01436597.2014.970870	100-149	Chapter 22

123.	Savell, E., Fooks, G., & Gilmore, A. B. (2016). How does the alcohol industry attempt to influence marketing regulations? A systematic review. Addiction, 111(1), 18–32. https://doi.org/10.1111/add.13048	250+	Chapter 2
124.	Savell, E., Gilmore, A. B., & Fooks, G. (2014). How Does the Tobacco Industry Attempt to Influence Marketing Regulations? A Systematic Review. PLoS ONE, 9(2), e87389. https://doi.org/10.1371/journal.pone.0087389	250+	Chapter 5
125.	Schram, A., Labonte, R., Baker, P., Friel, S., Reeves, A., & Stuckler, D. (2015). The role of trade and investment liberalization in the sugar-sweetened carbonated beverages market: A natural experiment contrasting Vietnam and the Philippines. Globalization and Health, 11(1), 41. https://doi.org/10.1186/s12992-015-0127-7	100-149	Chapter 24
126.	Scott, C., Hawkins, B., & Knai, C. (2017). Food and beverage product reformulation as a corporate political strategy. Social Science & Medicine, 172, 37–45. https://doi.org/10.1016/j.socscimed.2016.11.020	50-99	Chapter 2
127.	Sismondo, S. (2008). How pharmaceutical industry funding affects trial outcomes: Causal structures and responses. Social Science & Medicine, 66(9), 1909–1914. https://doi.org/10.1016/j.socscimed.2008.01.010	200-249	Chapter 30
128.	Smith, K. E., Fooks, G., Collin, J., Weishaar, H., & Gilmore, A. B. (2010). Is the increasing policy use of Impact Assessment in Europe likely to undermine efforts to achieve healthy public policy? Journal of Epidemiology & Community Health, 64(6), 478–487. https://doi.org/10.1136/jech.2009.094300	50-99	Chapter 17
129.	Smith, K. E., Fooks, G., Collin, J., Weishaar, H., Mandal, S., & Gilmore, A. B. (2010). "Working the System"—British American Tobacco's Influence on the European Union Treaty and Its Implications for Policy: An Analysis of Internal Tobacco Industry Documents. PLoS Medicine, 7(1), e1000202. https://doi.org/10.1371/journal.pmed.1000202	100-149	Chapter 8
130.	Smith, K. E., Savell, E., & Gilmore, A. B. (2013). What is known about tobacco industry efforts to influence tobacco tax? A systematic review of empirical studies. Tobacco Control, 22(2), e1–e1. https://doi.org/10.1136/tobaccocontrol-2011-050098	200-249	Chapter 11
131.	Smith, R. (2003). Medical journals and pharmaceutical companies: Uneasy bedfellows. BMJ, 326(7400), 1202–1205. https://doi.org/10.1136/bmj.326.7400.1202	250+	Chapter 8
132.	Steinman, M. A., Bero, L. A., Chren, M.-M., & Landefeld, C. S. (2006). Narrative Review: The Promotion of Gabapentin: An Analysis of Internal Industry Documents. Annals of Internal Medicine, 145(4), 284. https://doi.org/10.7326/0003-4819-145-4-200608150-00008	250+	Chapter 8
133.	Stockwell, T., Andreasson, S., Cherpitel, C., Chikritzhs, T., Dangardt, F., Holder, H., Naimi, T., & Sherk, A. (2021). The	50-99	seminal author

	burden of alcohol on health care during COVID -19. Drug and Alcohol Review, 40(1), 3–7. https://doi.org/10.1111/dar.13143		
134.	Stockwell, T., Zhao, J., Macdonald, S., Pakula, B., Gruenewald, P., & Holder, H. (2009). Changes in per capita alcohol sales during the partial privatization of British Columbia's retail alcohol monopoly 2003–2008: A multi-level local area analysis. Addiction, 104(11), 1827–1836. https://doi.org/10.1111/j.1360-0443.2009.02658.x	100-149	Chapter 10
135.	Stockwell, T., Zhao, J., Macdonald, S., Vallance, K., Gruenewald, P., Ponicki, W., Holder, H., & Treno, A. (2011). Impact on alcohol-related mortality of a rapid rise in the density of private liquor outlets in British Columbia: A local area multi-level analysis. Addiction, 106(4), 768–776. https://doi.org/10.1111/j.1360-0443.2010.03331.x	100-149	Chapter 10
136.	Stockwell, T., Zhao, J., Martin, G., Macdonald, S., Vallance, K., Treno, A., Ponicki, W., Tu, A., & Buxton, J. (2013). Minimum Alcohol Prices and Outlet Densities in British Columbia, Canada: Estimated Impacts on Alcohol-Attributable Hospital Admissions. American Journal of Public Health, 103(11), 2014–2020. https://doi.org/10.2105/AJPH.2013.301289	150-199	Chapter 10
137.	Stuckler, D., & Nestle, M. (2012). Big Food, Food Systems, and Global Health. PLoS Medicine, 9(6), e1001242. https://doi.org/10.1371/journal.pmed.1001242	250+	Chapter 9
138.	Stuckler, D., Basu, S., & McKee, M. (2011). Global Health Philanthropy and Institutional Relationships: How Should Conflicts of Interest Be Addressed? PLoS Medicine, 8(4), e1001020. https://doi.org/10.1371/journal.pmed.1001020	150-199	Chapter 22
139.	Stuckler, D., McKee, M., Ebrahim, S., & Basu, S. (2012). Manufacturing Epidemics: The Role of Global Producers in Increased Consumption of Unhealthy Commodities Including Processed Foods, Alcohol, and Tobacco. PLoS Medicine, 9(6), e1001235. https://doi.org/10.1371/journal.pmed.1001235	250+	Chapter 21
140.	Taillie, L. S., Reyes, M., Colchero, M. A., Popkin, B., & Corvalán, C. (2020). An evaluation of Chile's Law of Food Labeling and Advertising on sugar-sweetened beverage purchases from 2015 to 2017: A before-and-after study. PLOS Medicine, 17(2), e1003015. https://doi.org/10.1371/journal.pmed.1003015	250+	Chapter 14
141.	Tesler, L. E., & Malone, R. E. (2008). Corporate Philanthropy, Lobbying, and Public Health Policy. American Journal of Public Health, 98(12), 2123–2133. https://doi.org/10.2105/AJPH.2007.128231	150-199	Chapter 16
142.	Thow, A. M., Jones, A., Hawkes, C., Ali, I., & Labonté, R. (2017). Nutrition labelling is a trade policy issue: Lessons from an analysis of specific trade concerns at the World Trade Organization. Health Promotion International, daw109. https://doi.org/10.1093/heapro/daw109	100-149	Chapter 14

143.	Torchia, M., Calabrò, A., & Morner, M. (2015). Public–Private Partnerships in the Health Care Sector: A systematic review of the literature. Public Management Review, 17(2), 236–261. https://doi.org/10.1080/14719037.2013.792380	250+	Chapter 29
144.	Ulucanlar, S., Fooks, G. J., & Gilmore, A. B. (2016). The Policy Dystopia Model: An Interpretive Analysis of Tobacco Industry Political Activity. PLOS Medicine, 13(9), e1002125. https://doi.org/10.1371/journal.pmed.1002125		Chapter 2
145.	Ulucanlar, S., Fooks, G. J., Hatchard, J. L., & Gilmore, A. B. (2014). Representation and Misrepresentation of Scientific Evidence in Contemporary Tobacco Regulation: A Review of Tobacco Industry Submissions to the UK Government Consultation on Standardised Packaging. PLoS Medicine, 11(3), e1001629. https://doi.org/10.1371/journal.pmed.1001629	100-149	Chapter 11
146.	Van Schalkwyk, M. C. I., Petticrew, M., Cassidy, R., Adams, P., McKee, M., Reynolds, J., & Orford, J. (2021). A public health approach to gambling regulation: Countering powerful influences. The Lancet Public Health, 6(8), e614–e619. https://doi.org/10.1016/S2468-2667(21)00098-0	50-99	seminal author
147.	Vedula, S. S., Bero, L., Scherer, R. W., & Dickersin, K. (2009). Outcome Reporting in Industry-Sponsored Trials of Gabapentin for Off-Label Use. New England Journal of Medicine, 361(20), 1963–1971. https://doi.org/10.1056/NEJMsa0906126	250+	Chapter 19
148.	Wardle, C., & Singerman, E. (2021). Too little, too late: Social media companies' failure to tackle vaccine misinformation poses a real threat. BMJ, n26. https://doi.org/10.1136/bmj.n26	50-99	Chapter 20
149.	Watermeyer, R., Shankar, K., Crick, T., Knight, C., McGaughey, F., Hardman, J., Suri, V. R., Chung, R., & Phelan, D. (2021). 'Pandemia': A reckoning of UK universities' corporate response to COVID-19 and its academic fallout. British Journal of Sociology of Education, 42(5–6), 651–666. https://doi.org/10.1080/01425692.2021.1937058	100-149	GS search
150.	Weishaar, H., Dorfman, L., Freudenberg, N., Hawkins, B., Smith, K., Razum, O., & Hilton, S. (2016). Why media representations of corporations matter for public health policy: A scoping review. BMC Public Health, 16(1), 899. https://doi.org/10.1186/s12889-016-3594-8	50-99	Chapter 27
151.	West, D. M., Heith, D., & Goodwin, C. (1996). Harry and Louise Go to Washington: Political Advertising and Health Care Reform. Journal of Health Politics, Policy and Law, 21(1), 35–68. https://doi.org/10.1215/03616878-21-1-35	100-149	Chapter 5
152.	Wiist, W. H. (2006). Public Health and the Anticorporate Movement: Rationale and Recommendations. American Journal of Public Health, 96(8), 1370–1375. https://doi.org/10.2105/AJPH.2005.072298	100-149	Chapter 21
153.	Wood, B., Baker, P., & Sacks, G. (2021). Conceptualising the Commercial Determinants of Health Using a Power Lens: A	50-99	seminal author

	Review and Synthesis of Existing Frameworks. International Journal of Health Policy and Management, 1. https://doi.org/10.34172/ijhpm.2021.05		
154.	Yeh, J. S., Franklin, J. M., Avorn, J., Landon, J., & Kesselheim, A. S. (2016). Association of Industry Payments to Physicians With the Prescribing of Brand-name Statins in Massachusetts. JAMA Internal Medicine, 176(6), 763. https://doi.org/10.1001/jamainternmed.2016.1709	200-249	Chapter 8
155.	Yerger, V. B., & Malone, R. E. (2002). African American leadership groups: Smoking with the enemy: Table 1. Tobacco Control, 11(4), 336–345. https://doi.org/10.1136/tc.11.4.336	100-149	Chapter 5
156.	Yoon, S., & Lam, T.-H. (2013). The illusion of righteousness: Corporate social responsibility practices of the alcohol industry. BMC Public Health, 13(1), 630. https://doi.org/10.1186/1471-2458-13-630	150-199	Chapter 29
157.	Yue, R. P. H., Lee, H. F., & Wu, C. Y. H. (2017). Trade routes and plague transmission in pre-industrial Europe. Scientific Reports, 7(1), 12973. https://doi.org/10.1038/s41598-017-13481-2	50-99	Chapter 7
158.	Zhang, Q., Jiang, X., Tong, D., Davis, S. J., Zhao, H., Geng, G., Feng, T., Zheng, B., Lu, Z., Streets, D. G., Ni, R., Brauer, M., Van Donkelaar, A., Martin, R. V., Huo, H., Liu, Z., Pan, D., Kan, H., Yan, Y., … Guan, D. (2017). Transboundary health impacts of transported global air pollution and international trade. Nature, 543(7647), 705–709. https://doi.org/10.1038/nature21712	250+	Chapter 9
159.	Zhao, J., Stockwell, T., Martin, G., Macdonald, S., Vallance, K., Treno, A., Ponicki, W. R., Tu, A., & Buxton, J. (2013). The relationship between minimum alcohol prices, outlet densities and alcohol-attributable deaths in British Columbia, 2002–09. Addiction, 108(6), 1059–1069. https://doi.org/10.1111/add.12139	150-199	Chapter 10

Forward citation searching of seminal literature

Given the breadth of the CDOH research field covering numerous industries and diverse corporate sector practices (Gilmore et al., 2023), we identified seminal literature covering this breadth from a recently published book on the commercial determinants (Maani et al., 2022). Additionally, we identified more recently published 'seminal' articles by searching for seminal authors (individuals who authored multiple publications included as seminal literature), through a targeted Google Scholar search, and by including the Lancet Commission series on CDOH.

Identification of seminal literature from Maani et al., 2022

- One researcher screened the titles of all references in all book chapters, and if in doubt consulted abstracts and discussed unclear cases with a second researcher (week 23-25, 2024)
- Literature was included as 'seminal' if:
    - The reference was cited > 50 times (as per Scopus metrics), and

- it referred to the role of commercial entities in influencing health, and
- took a critical stance;
- Keywords for inclusion as 'seminal' are detailed below
- If article titles did not refer to health but were published in a public health relevant journal (e.g. American Journal of Public Health, The Lancet, BMC public health, Cochrane) they were included.

Keywords for commercial entities

- industry, corporate, commercial, transnational corporation, public private partnership, lobbying, advertising, marketing, consumer (only when focussing on industry, not individual consumer's health choice), trade, company, market, business, interest group, large cooperation name (coca cola, etc.), corruption, brand, packaging, representative, firms, sale, monopoly, privatization, outlet, influence on governments, sponsorship, labelling, pre-emption, supermarkets, private sector, conflict of interest, capitalism, public interest, financial interest, pricing (only when focussing on industry influence, not behavioural effects of pricings/taxes), think tanks, corporate social responsibility (external, internal only if major aspects of health, e.g. mental & physical health have been considered, not only happiness/loyalty to the employer)

Keywords for health and health-related topics

- gambling, firearms, alcohol, tobacco, sugar, processed food, cigarettes, smoking, breastfeeding, addiction, tanning, medication, medical/public health research, physicians, vaccine, pandemic, NCDs, pharma, Big (industry name, e.g. food, tobacco, pharma), disease, SDGs

Identification of further seminal literature (published from 2021 onwards)
(01/07/2024)

- Articles published in the **Lancet Commission series on commercial determinants of health** (if over 50 citations in Scopus)
- Relevant work (commercial AND health concepts) of **‚seminal authors'** who have first-authored three or more publications in the previously identified seminal literature; with 50 or more citations in Scopus: Baker, Phillip; Barlow, Pepita; Gilmore, Anna B.; Hawkins, Benjamin; Knai, Cécile; Maani, Nason; Mialon, Melissa; Petticrew, Mark; Smith, Katherine E.; Stockwell, Tim; and Stuckler, David.
- **Google Scholar search**
    - Search strategies:
        - *‚commercial determinant', health'* filtered 2021-2024 and most relevant (158 results, 3 relevant new results)
        - *‚commercial determinants' ‚health'* filtered 2021-2024 and most relevant (~2,900 results, first 20 pages have been screened, 0 relevant new results)
        - *‚commercial', health'* filtered 2021-2024 and most relevant (~770,000 results, first 20 pages have been screened, 0 relevant new results)
        - *‚corporate determinant' ‚health'* filtered 2021-2024 and most relevant (9 results, 0 relevant new results)
        - *‚corporate determinants', health'* filtered 2021-2024 and most relevant (257 results, first 20 pages have been screened, 0 relevant new results)
        - *‚corporate' ‚health',* filtered 2021-2024 and most relevant (~572,000 results, first 20 pages have been screened, 11 relevant new results)
    - with more than 50 citations in Scopus

This process resulted in identification of 159 seminal articles for forward citation searching.

Search strand 2: Database searching

Through database searches, we aim to identify articles that explicitly say "commercial determinants" or "CDOH", refer to the corporate sector practices, a key concept in CDOH research, or associated keywords; and are authored by individuals affiliated with German universities.

Overall search structure:

- CDOH
- AND German affiliation

Search building process:

- The search block for Germany / German was developed by a search specialist (Ina Monsef) and we were granted permission to use it in this project; it was double-checked by the search specialist who reviewed our searches
- Search terms for explicit mention of 'CDOH' were developed and subsequently adapted to ensure variations such as 'commercial and political determinants' would also be included
- CDOH concepts: we defined the corporate sector practices (Gilmore et al., 2023) and the mechanisms through which they influence health as core CDOH concepts.
  These include:
    - Political practices
    - Financial practices
    - Scientific practices
    - Marketing practices
    - Supply chain and waste practices
    - Labour and employment practices
    - Reputational management practices
- We operationalised the searches for these by building a search term around ((industry / commercial / corporate) adjacent (influence or synonyms)) AND (health or synonyms) (see below; e.g. OVID search, line 6)
- This search term included a number of synonyms to identify a wide range of articles; however, we had to use a restrictive 'adjacent' operator as opposed to AND or a broader 'adjacent' operator to limit the number of search results;
- We further identified keywords representing corporate strategies during our review of CDOH literature when identifying 'seminal' articles, e.g. lobbyism, corporate social responsibility, public-private partnership (see below, e.g. OVID search, line 7)
- We translated the OVID search to WoS by hand; for Google Scholar, we opted for only searching in German language.
- We assessed whether MesH terms matched our search and included 'commercial determinants of health/' in our OVID search. However, as it did not render any additional results we did not include it in the final search

Seed papers used to develop and test the search:

- https://doi.org/10.1007/s13679-023-00524-1
- https://dx.doi.org/10.1111/mcn.13632

Databases

Original searches displayed, results of search updates below original searches.

Medline and Embase via OVID

Search Date: July 29, 2024; 798 hits
Databases searched:

- Ovid MEDLINE(R) ALL 1946 to July 26, 2024
- Embase <1974 to 2024 July 26>

#	Searches	Results
1	(deutsch* or german*).in.	3,989,477
2	(Berlin* or Hamburg* or Mu?nchen* or Ko?ln* or Frankfurt* or Stuttgart* or Du?sseldorf* or Leipzig* or Dortmund* or Essen or Bremen* or Dresden* or Hannover* or Nu?rnberg* or Duisburg* or Bochum* or Wuppertal* or Bielefeld* or Bonn* or Mu?nster* or Mannheim* or Karlsruhe* or Augsburg* or Wiesbaden* or Mo?nchengladbach* or Gelsenkirchen* or Aachen* or Braunschweig* or Chemnitz* or Kiel* or Halle* or Magdeburg* or Freiburg* or Krefeld* or Mainz* or Lubeck* or Luebeck* or Erfurt* or Oberhausen* or Rostock* or Kassel* or Hagen* or Potsdam* or Saarbru?cken* or Hamm* or Oldenburg* or Mulheim* or Mulheim* or Osnabruck* or Osnabrueck* or Leverkusen* or Heidelberg* or Darmstadt* or Solingen* or Regensburg* or Herne* or Paderborn* or Neuss* or Ingolstadt* or Offenbach* or Furth or Fuerth* or Heilbronn* or Pforzheim* or Wurzburg* or Wuerzburg* or Wolfsburg* or Gottingen* or Goettingen* or Bottrop* or Reutlingen* or Erlangen* or Bremerhaven* or Koblenz* or Bergisch Gladbach* or Remscheid* or Trier* or Recklinghausen* or Jena* or Moers* or Salzgitter* or Siegen* or Gutersloh* or Guetersloh*or Hildesheim* or Hanau* or Kaiserslautern* or Cottbus* or Chosebuz* or Schwerin* or Witten* or Esslingen* or Ludwigsburg* or Gie?en* or Gera or Duren* or Dueren* or Tubingen* or Tuebingen* or Flensburg* or Iserlohn* or Villingen* or Ratingen* or Zwickau* or Lunen* or Luenen* or Konstanz* or Worms* or Marl or Minden* or Velbert* or Norderstedt* or Bamberg* or Dessau* or Ro?lau* or Neumunster* or Neumuenster* or Delmenhorst* or Viersen* or Rheine* or Marburg* or Luneburg* or Lueneburg* or Dorsten* or Troisdorf* or Wilhelmshaven* or Gladbeck* or Landshut* or Detmold* or Bayreuth* or Arnsberg* or Castrop Rauxel* or Brandenburg* or Aschaffenburg* or Bocholt* or Ludenscheid* or Luedenscheid* or Celle* or Kempten* or Fulda* or Lippstadt* or Aalen* or Dinslaken* or Herford* or Russelsheim* or Ruesselsheim* or Kerpen* or Neuwied* or Weimar* or Dormagen* or Sindelfingen* or Plauen* or Grevenbroich* or Rosenheim* or Neubrandenburg* or Friedrichshafen* or Herten* or Bergheim* or Schwabisch* or Schwaebisch* or Offenburg* or Garbsen* or Wesel* or Ulm* or Hurth* or Huerth* or Unna* or Langenfeld* or Euskirchen* or Greifswald* or Heidelberg* or Rhine or Neckar* or munich* or garmisch parten* or Freiburg* or Leipzig* or Bonn).in.	4,307,318
3	(nordrhein westfalen or westfalisch* or north rhine westphalia* or westphalia* or nrw* or ruhr or ruhrgebiet* or Bayern* or bavaria* or bayerisch* or Baden* or badisch* or Wu?rttember* or Niedersachsen* or niedersa?chs* or Saxon* or Hesse* or hessisch* or Sachsen* or sa?chsisch* or Rheinla?nd* or Pfalz* or pfa?lzisch* or Rhineland* or Palatinat* or Berlin* or Schleswig* or Holstein or holsteinisch* or Brandenburg* or Sachsen Anhalt* or sa?chsisch* or Thu?ringen* or thuringi* or thu?ringisch* or Hamburg* or Mecklenburg* or Vorpommer* or Pomerania* or Saarla?nd* or Bremen* or bremisch*).in.	1,018,477

4	1 or 2 or 3	4,745,512
5	(((commercial* or corporate) adj4 determinant*) or CDOH).ti,ab,kw,kf.	929
6	(((commercial* or corporat* or industr*) adj4 (practice* or interest* or power or influenc* or strateg* or tactic* or activit*)) and (health* or unhealthy or disease* or sick or well-being or wellbeing)).ti,ab,kw,kf.	24,461
7	((corporate social responsibilit* or marketing or advertis* or lobby* or industry sponsorship* or industry funding or conflict of interest or vested interest* or corruption or public?private partnership* or self-regulation) adj4 (health* or unhealthy or disease* or sick or well-being or wellbeing)).ti,ab,kw,kf.	12,088
8	5 or 6 or 7	36,707
9	4 and 8	1,590
10	limit 9 to "remove preprint records"	1,582
11	limit 10 to conference abstract status [Limit not valid in Ovid MEDLINE(R); records were retained]	784
12	10 not 11	**798**

Update:
CDOH search block (lines 5-7) complemented with another line:
((alcohol or tobacco or fossil fuel or pharma* or food or healthcare or gambling or chemical or tech* or financ* or mining or car or automotive) adj (industr* or company or companies or business or corporation) adj8 (health* or unhealthy or disease* or sick or well-being or wellbeing)).ti,ab,kw,kf.

Total of 1,243 hits (**417 references** exported after excluding old search)

Web of Science Core Collection

Search date: 29.07.2024; no of hits: 411
Indices searched:

- Science Citation Index Expanded (SCI-EXPANDED)--1900-present
- Social Sciences Citation Index (SSCI)--1990-present
- Arts & Humanities Citation Index (AHCI)--1990-present
- Emerging Sources Citation Index (ESCI)--2005-present

c	Searches	Results
1	OG=(german*)	198,590
2	OG=(nordrhein westfalen or westfalisch* or north rhine westphalia* or westphalia* or nrw* or ruhr or ruhrgebiet* or Bayern* or bavaria* or bayerisch* or Baden* or badisch* or Wu?rttember* or Niedersachsen* or niedersa?chs* or Saxon* or Hesse* or hessisch* or Sachsen* or sa?chsisch* or Rheinla?nd* or Pfalz* or pfa?lzisch* or Rhineland* or Palatinat* or Berlin* or Schleswig* or Holstein or holsteinisch* or Brandenburg* or Sachsen Anhalt* or	222,598

	sa?chsisch* or Thu?ringen* or thuringi* or thu?ringisch* or Hamburg* or Mecklenburg* or Vorpommer* or Pomerania* or Saarla?nd* or Bremen* or bremisch*)	
3	OG=(Berlin* or Hamburg* or Mu?nchen* or Koeln* or Frankfurt* or Stuttgart* or Du?sseldorf* or Leipzig* or Dortmund* or Essen or Bremen* or Dresden* or Hannover* or Nu?rnberg* or Duisburg* or Bochum* or Wuppertal* or Bielefeld* or Bonn* or Mu?nster* or Mannheim* or Karlsruhe* or Augsburg* or Wiesbaden* or Mo?nchengladbach* or Gelsenkirchen* or Aachen* or Braunschweig* or Chemnitz* or Kiel* or Halle* or Magdeburg* or Freiburg* or Krefeld* or Mainz* or Lubeck* or Luebeck* or Erfurt* or Oberhausen* or Rostock* or Kassel* or Hagen* or Potsdam* or Saarbru?cken* or Hamm* or Oldenburg* or Mulheim* or Mulheim* or Osnabruck* or Osnabrueck* or Leverkusen* or Heidelberg* or Darmstadt* or Solingen* or Regensburg* or Herne* or Paderborn* or Neuss* or Ingolstadt* or Offenbach* or Furth or Fuerth* or Heilbronn* or Pforzheim* or Wurzburg* or Wuerzburg* or Wolfsburg* or Gottingen* or Goettingen* or Bottrop* or Reutlingen* or Erlangen* or Bremerhaven* or Koblenz* or Bergisch Gladbach* or Remscheid* or Trier* or Recklinghausen* or Jena* or Moers* or Salzgitter* or Siegen* or Gutersloh* or Guetersloh*or Hildesheim* or Hanau* or Kaiserslautern* or Cottbus* or Chosebuz* or Schwerin* or Witten* or Esslingen* or Ludwigsburg* or Gie?en* or Gera or Duren* or Dueren* or Tubingen* or Tuebingen* or Flensburg* or Iserlohn* or Villingen* or Ratingen* or Zwickau* or Lunen* or Luenen* or Konstanz* or Worms* or Marl or Minden* or Velbert* or Norderstedt* or Bamberg* or Dessau* or Ro?lau* or Neumunster* or Neumuenster* or Delmenhorst* or Viersen* or Rheine* or Marburg* or Luneburg* or Lueneburg* or Dorsten* or Troisdorf* or Wilhelmshaven* or Gladbeck* or Landshut* or Detmold* or Bayreuth* or Arnsberg* or Castrop Rauxel* or Brandenburg* or Aschaffenburg* or Bocholt* or Ludenscheid* or Luedenscheid* or Celle* or Kempten* or Fulda* or Lippstadt* or Aalen* or Dinslaken* or Herford* or Russelsheim* or Ruesselsheim* or Kerpen* or Neuwied* or Weimar* or Dormagen* or Sindelfingen* or Plauen* or Grevenbroich* or Rosenheim* or Neubrandenburg* or Friedrichshafen* or Herten* or Bergheim* or Schwabisch* or Schwaebisch* or Offenburg* or Garbsen* or Wesel* or Ulm* or Hurth* or Huerth* or Unna* or Langenfeld* or Euskirchen* or Greifswald* or Heidelberg* or Rhine or Neckar* or munich* or garmisch parten* or Freiburg* or Leipzig* or Bonn)	820,002
4	1 or 2 or 3	1,009,233
5	TS=(CDOH OR ((commercial* OR corporate) NEAR/4 determinant*))	1,814
6	TS=(((commercial* or corporat* or industr*) NEAR/4 (practice* or interest* or power or influenc* or strateg* or tactic* or activit*)) and (health* or unhealthy or disease* or sick or well-being or wellbeing))	18,945
7	TS=((“corporate social responsibilit*” or marketing or advertis* or lobby* or “industry sponsorship*” or “industry funding” or “conflict of interest” or “vested interest*” or corrupt* or “public-private partnership*” or self-regulation) NEAR/4 (health* or unhealthy or disease* or sick or well-being))	17,214
8	5 or 6 or 7	37,261
9	4 and 8	**411**

Update:
CDOH search block (lines 5-7) complemented with another line:

TS=(((alcohol or tobacco or fossil or pharma* or food or healthcare or gambling or chemical or tech* or financ* or mining or car or automotive) NEAR/0 (industr* or company or companies or business or corporation)) NEAR/8 (health* or unhealthy or disease* or sick or well-being))

Total of 532 hits (**104** references exported after excluding old search)

Google Scholar

Search date: 29.07.2024, no of hits: 50

c	Searches	Results
1	"kommerzielle determinante" "gesundheit"	2
2	"kommerzielle determinanten" "gesundheit"	12
3	"kommerziellen determinante" "gesundheit"	1
4	"kommerziellen determinanten" "gesundheit"	25
5	"kommerzielle gesundheitsdeterminante"	2
6	"kommerzielle gesundheitsdeterminanten"	3
7	“kommerziellen gesundheitsdeterminante"	0
8	"kommerziellen gesundheitsdeterminanten"	5
	gesamt, incl Duplikate	50

- http://scholar.google.de/ncr
- Search specifications for advanced search:
- anytime, anywhere in the article
- Sorted as most relevant
- Excluding citations

Screening of first 10 pages (100 results) immediately after conducting the search; taking screenshots of results as a measure of replication

Update - GS not included in update
