# Supplementary material for: Commercial determinants of health—a scoping review of research ‘made in Germany’
Source: Eur J Public Health. 2026 Mar 17;36(2):ckag030. doi: 10.1093/eurpub/ckag030 (PMC13017704; doi:10.1093/eurpub/ckag030)
Supplement: ckag030_Supplementary_Data [file ckag030_supplementary_data.zip › ejph-2025-04-om-0262-File007.pdf]

Additional file 3: Methods appendix

Eligibility

Faced with diverse bodies of literature linked to the seven corporate sector practices we used general eligibility criteria as outlined in our protocol and manuscript and more detailed criteria for the general eligibility criteria, in particular for specific corporate sector practices.

- **Peer review**: We faced difficulties to determine if certain articles were peer-reviewed, which we addressed as follows: We reviewed if peer review reports were available and assessed article time stamps (e.g. “submitted XX.XX.XXXX, accepted XX.XX.XXXX”) to ascertain if peer-review had taken place. We further reviewed journals’ peer review policies. As the peer review status for letters and editorials remained difficult to ascertain we decided post-hoc to exclude those.
- **Critical examination of corporate sector practices and their effects on the political, economic, and social systems affecting health**
  - Exclude where population health is only peripherally concerned or not mentioned at all
  - Exclude where a specific industry is only peripherally concerned or not mentioned at all, e.g.:
    - A study on smoking prevalence in France without mentioning industry
    - Studies on air pollution and population health without mentioning responsible industry/industries
    - Studies examining ambient air pollution, PM 2.5, toxin levels in soil or water without mentioning responsible industry/industries
  - Exclude articles that counter commercial influence or act as measures <u>against</u> corporate sector practices, e.g. research evaluating sugar taxes or testing the acceptability of tobacco control measures
  - Occupational health:
    - exclude when the main focus of the article is on workplace health promotion
    - exclude articles examining workplace prevalence of certain diseases without mentioning workplace health as a systemic or structural aspect linked to corporate sector labour and employment practices
  - Exclude articles that evaluate public-private partnerships in a non-critical manner.
  - Exclude articles exclusively authored by individuals with industry affiliations as not taking critical stance
  - Exclude articles sponsored by industry as not taking critical stance

Data extraction

With regards to **corporate sector practices**, we used examples of practices outlined by Gilmore and colleagues (2023). For practices which were not included in their examples we established the most appropriate corporate sector practice through team discussion. Some practices fit

multiple categories of corporate sector practices: For examples, industry self-regulation practices were coded as political, financial and reputational practices; and bribery of healthcare workers by pharmaceutical industry was coded as financial, scientific and marketing practices.

In order to determine the **article type**, we distinguished between ‘primary’ and ‘secondary’ original research, ‘opinion pieces’, ‘overviews’, ‘theoretical work’ and ‘other’. Primary original research was categorized into quantitative, qualitative and mixed methods. We marked research as mixed, when for example a qualitative analysis of marketing strategies was performed, and their usage patterns were analysed with quantitative methods. Reviews constitute original research when they are based on systematic searches in a minimum of two databases and outline rigorous processes for screening, eligibility, and analysis. Overviews and opinion pieces do not provide a systematic search strategy but the tone in overviews is more formal and objective than in opinion pieces and draws more systematically on relevant literature. We furthermore differentiated theoretical work (e.g. originated from political sciences or developing the CDOH concept). All articles not fitting these categories were labelled as ‘other’.

To determine the **scientific discipline,** we considered a) the first author’s institutional affiliation, b) the journal and c) the overall article scope and when possible created sub-categories (e.g. public health nutrition or occupational and environmental public health).

To retrieve information on funding and COI we searched the full texts. We coded the funding category as ‘public’ if funding came from a state organisation; and as ‘charity’ if funding came from a private organisation (e.g. trusts, foundations), which we ascertained through review of funders’ homepages.

Table S3.1: Coding frame for data extraction

Category	Codes	Code description	Example
Corporate sector practices	*Marketing practices*	“Practices to promote sales of products or services“ (1)	Influence of visits/contacts of pharmaceutical sales representatives on doctors’ drug prescriptions (2)
	Scientific practices	“Practices involving the production and use of science to alter products or otherwise secure favourable outcomes (or both) for the industry” (1)	Investigation how Food/Beverages Industry sponsorship of systematic reviews on health effects of sugar-sweetened beverages shapes conclusions (3)
	Political practices	“Practices to secure preferential treatment or prevent, shape, circumvent, or undermine public policies (or a combination of the above) in ways that further corporate interests” (1)	Big Tech’s lobbying strategies to avoid governmental regulation & their effects on health (4)

	Reputational management practices	“Efforts to shape legitimacy and credibility, reduce risk, and enhance corporate brand image“ (1)	Investigation into Alcohol industries’ pledge to combat drink-driving via mass media campaigns and partnerships with road safety NGOs (5)
	Financial practices	“Practices to support financial position of the organisation“ (1)	Usage of medical care centres in Bavaria as financial assets & tax avoidance (6)
	Supply chain & waste management practices	“Practices involved in the creation, distribution, retail, and waste management of products or services” (1)	Respiratory health outcomes of Chilean children living next to industrial gold or copper mines (7)
	Labour & employment practices	“Practices to manage people employed directly within, or under contract to, the organisation within its supply chain“ (1)	Effects of working in moderate cold environments within producing food/beverage industries (8)
Article type	*Original, primary research: quantitative*	*Methods:* Quantitative study designs, statistical analysis	“In a retrospective 2-armed cohort study, the prescription behavior of 6,996 German physicians, of which 2,354 had participated in at least 1 of 24 NIPMSs and 4,642 were controls, was analyzed.” (9)
	Original, primary research: qualitative	*Methods:* Qualitative study design, e.g. case studies, thematic analysis, document analysis	“We analyzed previously secret tobacco industry documents in the University of California, San Francisco Legacy Tobacco Documents Library, and IOM public access files.” (10)
	Original, primary research: mixed	*Methods:* Qualitative & quantitative	Qualitative analysis of advertisements within children’s TV programme + quantitative analysis of patterns, frequency, etc: “[…] television program of ten German television networks was recorded on a weekday and a weekend day. Data was analyzed using content and regression analysis.” (11)
	Original research: review	*Methods:* At least 2 data base searches & rigorous screening, eligibility and analysis processes reported	“We conducted a search of the PubMed, Cochrane Library, and Scopus databases […] on the association between SSB consumption and weight gain or obesity. SR conclusions were independently classified by two

			researchers into two groups […]. These two reviewers were blinded with respect to the stated source of funding and the disclosure of conflicts of interest.” (3)
	Overview	*Methods:* Unsystematic search strategy<br>*Tone:* Formal & objective<br>*Cited literature:* Systematic use of relevant literature	“The objective of this article is to provide a review of the historic background behind the pandemic tragedy of asbestos disease by focussing on the German experience.” (12)
	Opinion piece	*Methods:* Unsystematic search strategy<br>*Tone:* Opiniated	“One must have an understanding of the attempts of the interested parties to exercise some influence in the political sector, but at the same time there should be consensus that science should always be independent and uninfluenced.” (13)
	Theoretical work	*Content:* Advancing/Applying concept/framework	“Drawing on Donella Meadows’s systems thinking framework, this article examines how a systems perspective may be used to analyze the commercial determinants of NCDs […].” (14)
Scientific discipline	*Public Health*	First author’s affiliation & Journal & Article scope	*Affiliation:* “Center for Tobacco Control Research and Education, Department of Medicine”<br>*Journal:* “PLOS Medicine”<br>*Scope:* Focussing on BIG tobacco’s strategies to avoid governmental regulation (10)
	Healthcare	First author’s affiliation & Journal & Article scope	*Affiliation:* “Department of Psychiatry and Psychotherapy, University Medical Centre [City]”<br>*Journal:* “PLOS One”<br>*Scope:* Influence of pharmaceutical representatives on physicians’ drug prescription patterns (2)
	Public Health Nutrition	First author’s affiliation & Journal & Article scope	*Affiliation:* “Institute for Food and Resource Economics”<br>*Journal:* “Food Policy”<br>*Scope:* Focussing on unhealthy foods TV commercials aimed at children & relationship to industry self-regulation (11)
Funding & COI	*public*	Funding & COI statements within full texts &	“[Author] is funded by a NHS Research Scotland Senior Clinical Fellowship […]” (14)

		webpages of organisations in question	
	charity	Funding & COI statements within full texts & webpages of organisations in question	“[Author] is member of the ‘‘no free lunch’’ organization MEZIS e.V. in Germany.” (2)

Data charting and analysis: Social Network Analysis

In VOSviewer, we undertook the following steps to analyse co-authorship networks.

File >> Create >> Create a map based on bibliographic data >> Read data from reference manager files (using a RIS file of all 136 included records)

- *Type of analysis*: co-authorship, *unit of analysis*: author, *counting method*: full counting; not ignoring documents with a large number of authors
- *Threshold*: minimum number of documents of an author: 2
- Selecting network, including all clusters and authors

References:

1. Gilmore AB, Fabbri A, Baum F, Bertscher A, Bondy K, Chang HJ, et al. Defining and conceptualising the commercial determinants of health. The Lancet. 2023 Apr 8;401(10383):1194–213.

2. Lieb K, Scheurich A. Contact between doctors and the pharmaceutical industry, their perceptions, and the effects on prescribing habits. PLoS ONE. 2014;9(10):e110130.

3. Bes-Rastrollo M, Schulze MB, Ruiz-Canela M, Martinez-Gonzalez MA. Financial Conflicts of Interest and Reporting Bias Regarding the Association between Sugar-Sweetened Beverages and Weight Gain: A Systematic Review of Systematic Reviews. PLOS Med. 2013;10(12).

4. Lock I, Davidson S. Argumentation strategies in lobbying: toward a typology. J Commun Manag. 2024;28(2):345–64.

5. Stein I, Bachani AM, Hoe C. The alcohol industry’s involvement with road safety NGOs. Glob Health [Internet]. 2022;18(1). Available from: https://www.scopus.com/inward/record.uri?eid=2-s2.0-85124679811&doi=10.1186%2fs12992-022-00813-9&partnerID=40&md5=e1b276be3451912fb6476e7be48f0642

6. Buzek R, Scheuplein C. The global wealth chains of private-equity-run physician practices. Tijdschr VOOR Econ EN Soc Geogr. 2022;113(4):331–47.

7. Herrera R, Radon K, Von Ehrenstein OS, Cifuentes S, Munoz DM, Berger U. Proximity to mining industry and respiratory diseases in children in a community in Northern Chile: A cross-sectional study. Environ Health Glob Access Sci Source. 2016;15(1):66.

8. Griefahn B, Mehnert P, Brode P, Forsthoff A. Working in moderate cold: A possible risk to health. J Occup Health. 1997;39(1):36-EP-44.

9. Koch C, Schleeff J, Techen F, Wollschläger D, Schott G, Kölbel R, et al. Impact of physicians' participation in non-interventional post-marketing studies on their prescription habits: A retrospective 2-armed cohort study in Germany. PLoS Med [Internet]. 2020;17(6). Available from: https://www.scopus.com/inward/record.uri?eid=2-s2.0-85087154962&doi=10.1371%2fjournal.pmed.1003151&partnerID=40&md5=ce05d7cc78e7afff3d25c626c01279c1

10. Tan CE, Kyriss T, Glantz SA. Tobacco Company Efforts to Influence the Food and Drug Administration-Commissioned Institute of Medicine Report Clearing the Smoke: An Analysis of Documents Released through Litigation. PLoS Med. 2013;10(5):e1001450.

11. Landwehr SC, Hartmann M. Industry self-regulation of food advertisement to children: Compliance versus effectiveness of the EU Pledge. Food Policy [Internet]. 2020;91. Available from: https://www.scopus.com/inward/record.uri?eid=2-s2.0-85078873790&doi=10.1016%2fj.foodpol.2020.101833&partnerID=40&md5=91e9493e7881f6efdf6e4210b72e481b

12. Baur X. Asbestos-related disorders in Germany: Background, politics, incidence, diagnostics and compensation. Int J Environ Res Public Health [Internet]. 2018;15(1). Available from: https://www.scopus.com/inward/record.uri?eid=2-s2.0-85040917630&doi=10.3390%2fijerph15010143&partnerID=40&md5=59049f8e5dc6ddf70583f0c7b95ec25c

13. Batra A. Funding support - Cui bono? Addiction. 2007;102(7):1035.

14. Knai C, Petticrew M, Mays N, Capewell S, Cassidy R, Cummins S, et al. Systems Thinking as a Framework for Analyzing Commercial Determinants of Health. Milbank Q. 2018;96(3):472–98.
